# Generating the polymorph landscapes of amyloid fibrils using AI: RibbonFold

**DOI:** 10.1073/pnas.2501321122

**Published:** 2025-04-15

**Authors:** Liangyue Guo, Qilin Yu, Di Wang, Xiaoyu Wu, Peter G. Wolynes, Mingchen Chen

**Affiliations:** ^a^Changping Laboratory, Beijing 102206, China; ^b^Center for Theoretical Biological Physics, Rice University, Houston, TX 77005; ^c^Department of Chemistry, Rice University, Houston, TX 77005; ^d^Department of Physics and Astronomy, Rice University, Houston, TX 77005; ^e^Department of Biosciences, Rice University, Houston, TX 77005

**Keywords:** polymorphism, amyloid, structure prediction, RibbonFold, AI

## Abstract

The concept that proteins are selected to fold into a well-defined native state has been effectively addressed within the framework of energy landscapes, underpinning the recent successes of structure prediction tools like AlphaFold. The amyloid fold, however, does not represent a unique minimum for a given single sequence. While the cross-*β* hydrogen-bonding pattern is common to all amyloids, other aspects of amyloid fiber structures are sensitive not only to the sequence of the aggregating peptides but also to the experimental conditions. This polymorphic nature of amyloid structures challenges structure predictions. In this paper, we use AI to explore the landscape of possible amyloid protofilament structures composed of a single stack of peptides aligned in a parallel, in-register manner. This perspective enables a practical method for predicting protofilament structures of arbitrary sequences: RibbonFold. RibbonFold is adapted from AlphaFold2, incorporating parallel in-register constraints within AlphaFold2’s template module, along with an appropriate polymorphism loss function to address the structural diversity of folds. RibbonFold outperforms AlphaFold2/3 on independent test sets, achieving a mean TM-score of 0.5. RibbonFold proves well-suited to study the polymorphic landscapes of widely studied sequences with documented polymorphisms. The resulting landscapes capture these observed polymorphisms effectively. We show that while well-known amyloid-forming sequences exhibit a limited number of plausible polymorphs on their “solubility” landscape, randomly shuffled sequences with the same composition appear to be negatively selected in terms of their relative solubility. RibbonFold is a valuable framework for structurally characterizing amyloid polymorphism landscapes.

## Significance

Despite advances in understanding protein folding, much less is known about amyloid formation, which involves complex mechanisms and polymorphism, where the same protein sequence can form structurally distinct fibrils. In this study, we introduce RibbonFold, an AI-driven model that incorporates amyloid-specific constraints to predict amyloid fibril structures. By combining the ribbon hypothesis with a curated amyloid database, RibbonFold accurately predicts polymorphic amyloid structures and explores their diverse landscape. This approach opens avenues for understanding amyloid aggregation and identifying potential therapeutic targets.

Evolution has sculpted the energy landscapes of many individual proteins into funnels so as to allow them to fold into reasonably well-organized structures, which can function in the cell (1, 2). The conformational ensembles of many proteins however are rather diverse, nevertheless allowing them to function while being deemed “intrinsically disordered” or “fuzzy” (3, 4). In addition, at sufficiently high concentrations in vitro or in vivo, peptides and even foldable proteins often aggregate, in rather specific ways. A common type of aggregation, domain swapping, actually arises from the evolved funneled nature of the monomer landscape which has allowed the monomers to fold productively. In domain swapping, native-like interactions paralleling those necessarily found in the monomer but involving distinct units, allow the concatenation of many largely folded units by intertwining their parts (5, 6). Surprisingly, there is another form of aggregation largely unrelated to the monomer structure and thus to the evolved aspects of the folding funnel: the formation of amyloids (7–9). Amyloids are a class of protein aggregates having, in part, highly ordered, fibrillar structures (10). These aggregates dramatically came to scientists’ attention first in the observation of prion diseases (11, 12) accompanied by neuro-degeneration such as Kuru and Scrapie. Amyloids are also known to be associated with a still wider range of diseases, including Alzheimer’s, Parkinson’s, and Type II diabetes (13). Amyloids also feature in models of memory formation (14–17) and amyloids have been shown to have positive functional roles in yeast (18). Recently amyloids have been found to be catalysts (19), although not yet as effective as natural enzymes.

Much less is understood about the molecular details of amyloid formation than about productive folding to the native state of monomers which is guided by the funnel and for which native contact formation dominates. The mechanisms of amyloid formation have several steps including primary nucleation (20), aggregate growth by monomer addition and secondary nucleation (21–23). These all involve many copies of the protein monomers. The molecular details of these steps do not seem to be strongly selected by sequence evolution, thus leading to many surprises. The initial seeds of primary nucleation seem to be small peptide fragments of the larger protein capable of forming strong associations (8, 24). Energetic analysis using coarse-grained energy function suggests only about half of all protein sequences possess such strongly associating amyloidogenic fragments (25). The growth of amyloids also has been shown to sometimes exhibit “backtracking” in which part of the growing structure must be pulled back before sequential aggregation can continue (26), indicating a frustrated, rugged nature of the aggregate growth landscape (27). Growth is also often intermittent (28). Nevertheless, many of these mechanistic steps in specific cases have become understood using the same coarse-grained force fields that have allowed the prediction of monomer structures of well-folded proteins and native folding mechanisms (16, 28–31).

In this paper, we explore the use of an AI method for finding candidates for stable amyloid structures employing neural network architectures like those employed by AlphaFold2 to predict the structures of natively folded monomers (32). Much of the success of AlphaFold2 in predicting biomolecular structures has come from the integration of coevolution data as in other (33, 34) prediction schemes but using attention networks as a computational framework. Since sequence evolution seems less controlling for amyloids, predicting the structures of amyloids across their polymorphic forms should be a distinct challenge. Nevertheless clearly there are physico-chemical constraints on the aggregation of proteins into ordered structures of the fibrils as opposed to amorphous aggregates which may have a near continuum of structures, giving hope that machine learning can help.

The most significant difficulty lies with the polymorphism of amyloids: it has been established that the same protein sequence can fold into multiple structurally distinct fibril forms (35). This multiplicity is a critical factor in the pathogenicity and possibly in the progression of amyloid-related diseases. Presumably the choice of polymorph depends on historical aspects of the formation process whether in vivo or in vitro. A long standing mystery of amyloid diseases is what determines their ages of onset (36). The stochastic evolution within the body of different polymorphs transforming into each other through secondary nucleation may explain the long timescale of the onset of disease progression which is otherwise so puzzling (37). The structural diversity of amyloid fibrils, even for a single protein, in any case complicates the development of accurate predictive models using neural networks based on empirical structural data alone (20). Some structures of several of multiple polymorphs of several amyloids have been inferred through difficult and painstaking work using solid state NMR, and cryo-EM methods (38–41). Nevertheless the number of the publicly available amyloid structures is small, in comparison to the over 200K folded protein entries in the PDB.

Understanding amyloid polymorphism is essential not only for unraveling the mechanisms of aggregation diseases but also for developing targeted therapeutic strategies for these diseases (42, 43). The challenge of predicting structures of amyloid is thus twofold. First, the complex and heterogeneous nature of amyloid fibrils makes it difficult for purely data-based machine learning to generate accurate structural models. Second, while amyloid fibrils exhibit a high degree of molecular order within their *β*-sheet-rich cores, their overall structures generally vary significantly depending on factors such as the environment in which they are formed, small sequence variations, and posttranslational modifications. This structural diversity is not easily captured by most current protein structure prediction tools, which have typically been focusing on predicting a single native fold of soluble proteins. The cross-*β* features also lead to quite glassy energy landscapes and thus slow dynamics impeding direct molecular dynamics.

We see then, that despite its notable success with well-folded proteins, AlphaFold2 faces significant challenges when applied to amyloids (44, 45). One way forward is to augment the training datasets used by AlphaFold2 with amyloid-specific data, including experimentally determined structures of amyloid fibrils and their polymorphs. Focusing on these cases by itself should improve the model’s ability to recognize and predict the *β*-sheet-rich architectures characteristic of amyloids. In the present paper, however, in addition, we use our knowledge of the physics of forming amyloids to constrain the prediction of fibril polymorphs to preserve their nearly universal energetically favored parallel-in-register *β* hydrogen bonding. Under the assumption of the dominance of this simple organization principle, we constrain the neural network to examine several monomeric chains arranged in a stack so that the assembly resembles a ribbon. We have taken this constraint approach to amyloid predictions earlier to predict polymorphic amyloid structures using molecular dynamics of a coarse-grained energy function (37). Under the parallel stacking constraint assumption, possible polymorphs of the fiber correspond to different two-dimensional folds of ribbons rather than arbitrary three-dimensional structures. In this way, we see that understanding the landscape of possible polymorphs can be looked at as a protein folding problem, not in three dimensions but rather one in two dimensions: the two-dimensional folding of polypeptides constrained to be ribbons. Under the ribbon hypothesis, the meanders of the ribbon and the locations of the contacting pleats of the ribbon are, at least initially, determined by the same solvent-averaged forces that determine the tertiary folds of all globular proteins. This approach in the coarse-grained dynamics framework has already proved quite successful for *Aβ* (37).

**Fig. 1.**
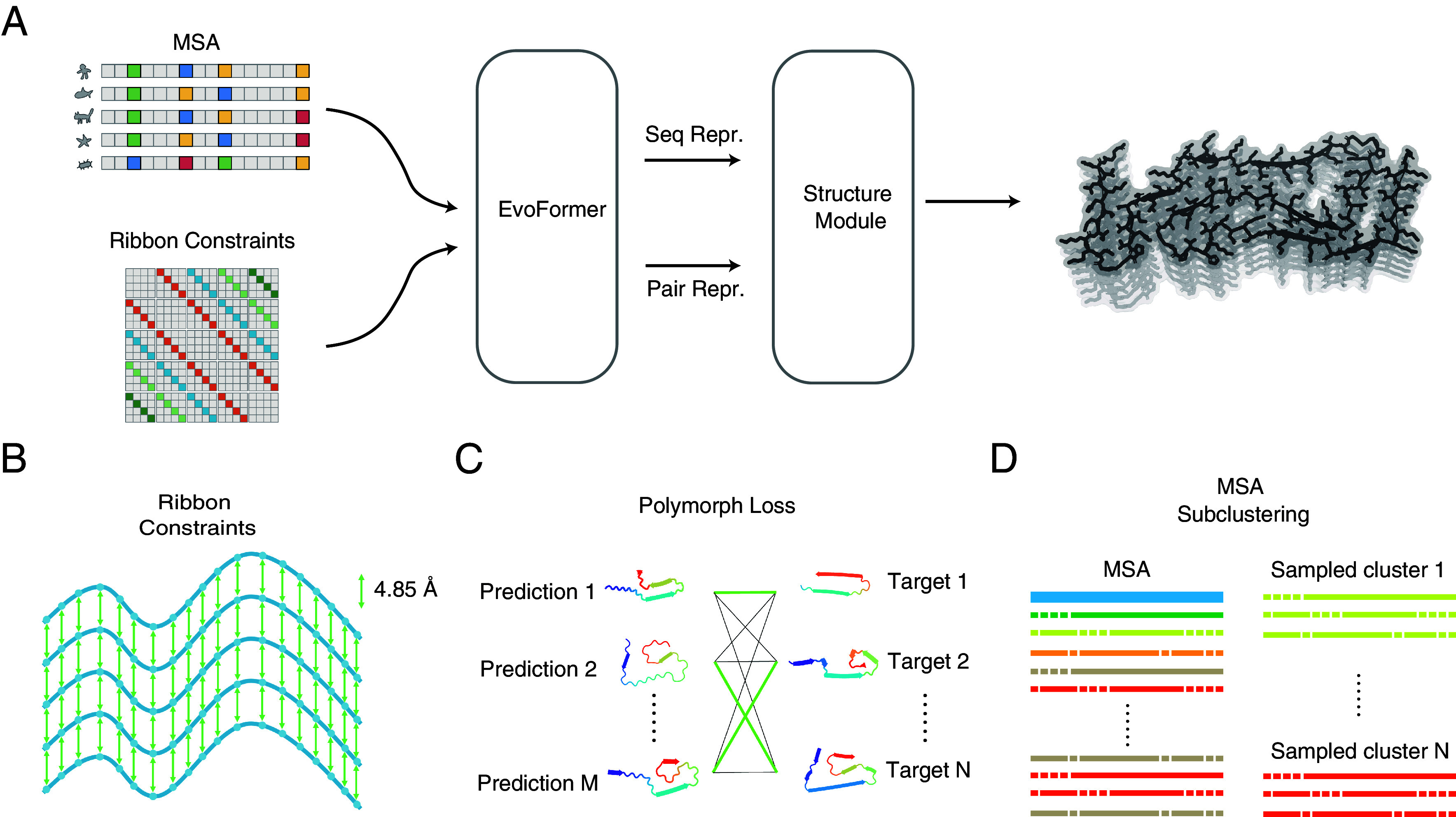
Schematic diagram of RibbonFold. (*A*) RibbonFold is adapted from the approach of AlphaFold2-Multimer (46). (*B*) The first major difference from AlphaFold2 is the modification of the template trunk to read in parallel-in-register ribbon constraints. For each residue from a chain, its contact with neighborhood residues in the same registry is encoded, while no intrachain contacts are added. In order to enhance the sampling efficiency in the structure module, the initial model is already an extended pentameric ribbon so that the simulated folding happens nearly on 2D. With the ribbon module, the parameters were finetuned on a curated dataset of around 500 monomeric amyloid structures. (*C*) Because many amyloid proteins are polymorphic, the N-to-M loss is designed to account for multiplicity of configurations for a given amyloid sequence during training. During training, the network will generate N predictions for each input sequence, find the best matched polymorph of this given sequence and back propagate the average of the summed values. (*D*) Default AlphaFold2 can only generate sampled structures with limited structural diversity. RibbonFold adopted MSA subclustering after obtaining the MSAs and carried out the predictions with subclusters to enhanced structural diversity (47).

In this paper, we also exploit the idea of the ribbon polymorph landscape, to inject ribbon constraints into AlphaFold2 as the template trunk (Fig. 1) and finetune the model using a curated database of amyloids (see *Materials and Methods* for details). The resulting model, which we call RibbonFold, predicts structures of amyloid fibrils with much higher accuracy than either AlphaFold2 or AlphaFold3 (TM score of 0.50). RibbonFold is also trained to generate diverse structural predictions for each given amyloid sequence, thus enabling exploration of the polymorph landscape of ordered fibrils. RibbonFold will potentially facilitate the search for druggable targets and possible enzyme activity.

For specific illustration of these ideas in this paper, we will focus on only a few paradigm systems: peptides derived from A*β*, Tau, and *α*-Synuclein, all of which are known to have polymorphic amyloid landscapes (48–50). Surprisingly, only a modest number of polymorphs come out of the structure prediction runs for the ribbon monomers. This paucity of structures is consistent with experimental observations. When the A*β* sequences are randomized however, the predicted amyloid structures turn out to be more diverse and individually less favorable energetically. The fact that all of the polymorphs of the scrambled sequence are less stable than those of the natural A*β* sequence suggests that there is a form of selection going on, which is distinct from the sequence selection needed for monomer folding. We believe this selection represents the constraint that in order for the amyloids to give rise to diseases, they must be able to form aggregates easily that are stable at cellular concentrations of protein (51). In fact, the limited multiplicity of amyloid polymorphs, generated by the sampling predictions on both the proteins from the training dataset as well as for sequences not in the training set, suggests that the amyloid landscapes while different from globular protein landscapes, are still funneled but imperfectly, thereby leading to the relatively small multiplicity of polymorphs.

## Significant Improvements in the Prediction of Amyloids

The performance of RibbonFold was evaluated against the performance of AlphaFold3 (52), AlphaFold2-Multimer, and AlphaFold2-Multimer with parallel-in-register constraints. The method “AlphaFold2 with parallel-in-register constraints” refers to simply introducing parallel-in-register constraints defined in our model, but using the original unmodified AlphaFold2-Multimer parameters that were learned mostly on globular proteins. AlphaFold3 results were obtained using the default AlphaFold3 settings. For AlphaFold2, the inference settings are the same as were used for RibbonFold. Two test sets were used in this evaluation. The first set was a subset derived from the dataset introduced above, containing 15 ribbons: 5 are distinct in sequence, which means their assigned sequence clusters are not shown in the training set; the other 10 are ribbons with sequence clusters seen in the training set but belonging to new structural clusters. To evaluate fairly the performance of RibbonFold, a second set was assembled as an independent test set which consists of four recently released ribbons whose sequences are novel, available after September 2024.

**Fig. 2.**
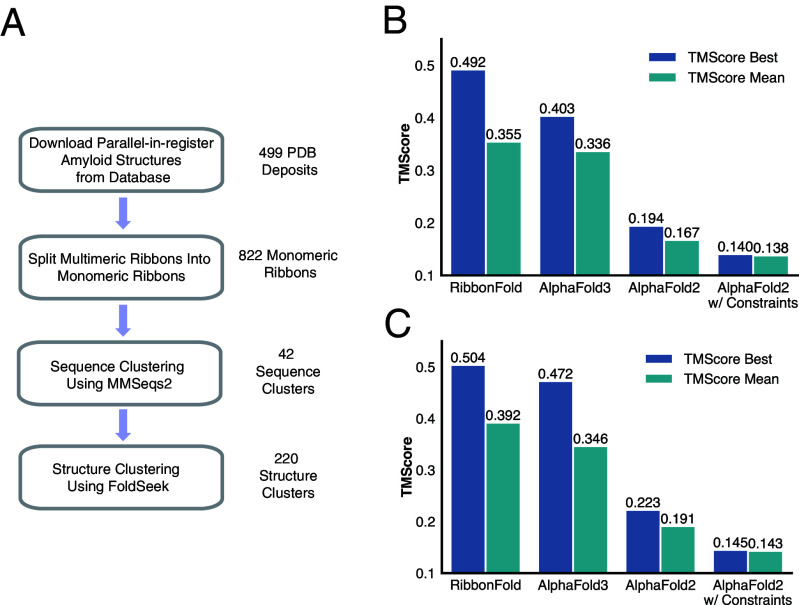
RibbonFold significantly outperforms AlphaFold2-Multimer and AlphaFold3 on independent datasets. (*A*) The training dataset was generated during data curation, containing amyloid structures deposited before 2024 Aug/31st. This test dataset is split by sequence homology and structural similarity to avoid data leakage. (*B*) The performance of RibbonFold is significantly better than AF2 and AlphaFold3 when we carry out 10 predictions for each input amyloid sequence on the test dataset curated during training. (*C*) The second test dataset is curated from amyloid structures deposited after Aug/31st. The performance of RibbonFold is significantly better than AF2 and AlphaFold3 when we carry out 10 predictions for each input amyloid sequence.

**Fig. 3.**
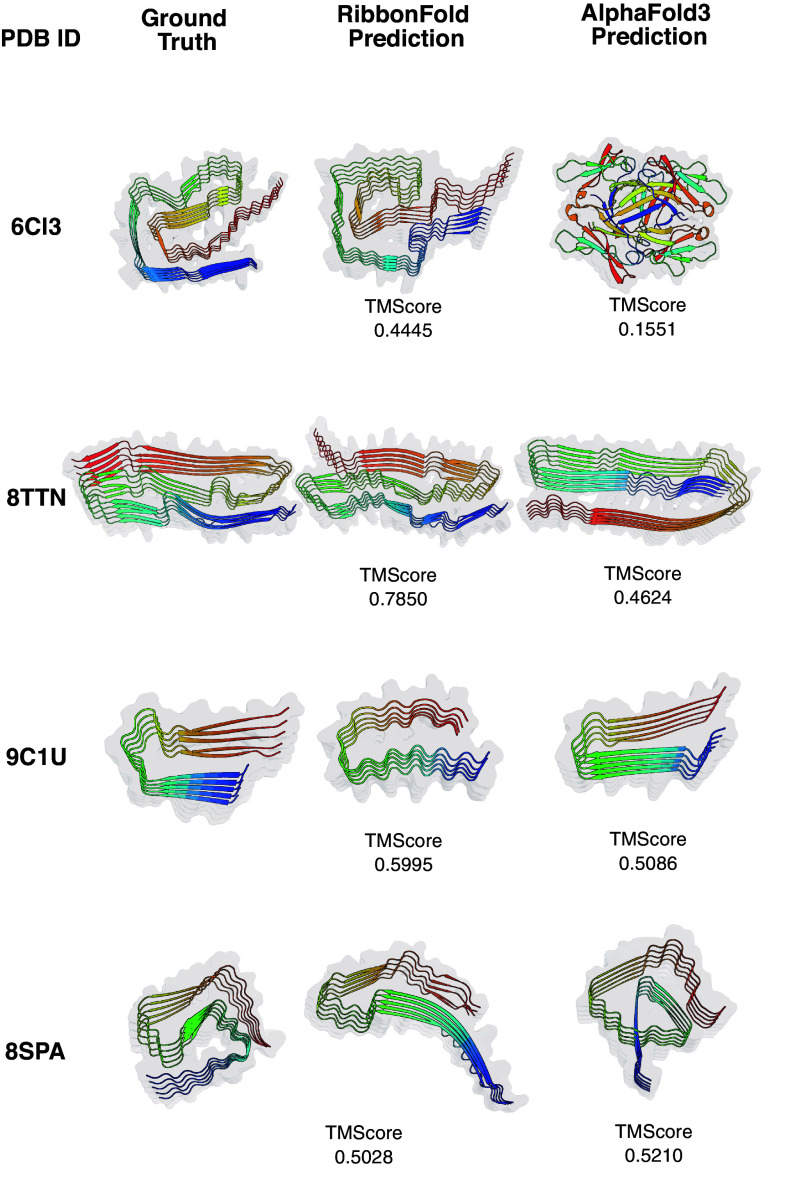
Example predictions from RibbonFold on the test set. For Amyloid derived from antibody L chain (PDB ID: 6CI3), AlphaFold3 did not capture the amyloid form with random loops entangled together. For Amyloid derived from Tau (PDB ID: 8TTN), while AlphaFold3 predicted the parallel-in-register fold, but missed the tertiary fold. RibbonFold accurately predicted the tertiary *β* fold in comparison with solved structures (color in white). Each chain in the ribbon was colored from blue (N termini) to red (C termini).

For each ribbon from the test set, 10 samples were generated for each method. We computed the TM-Score between each predicted sample and the corresponding ground truth structure, evaluating both the best TM-Score and the average TM-Score across the 10 samples. The best TM-Score out of ten predictions is used as the major metric, because most known amyloids are polymorphic and only the best score would reasonably address such polymorphism. A best TM-Score of 0.5 is already a reasonable structure in comparison with the solved natural polymorphs. Our results demonstrate that RibbonFold achieves greater accuracy in predicting ribbon structures compared to other approaches (Fig. 2 *B* and *C*). Notably, for 9 out of the 15 ribbons in the first dataset, RibbonFold achieved a 10-run best TM-Score at least 0.1 higher than AlphaFold3-Server (Fig. 2*B*). Fig. 3 presents selected examples comparing RibbonFold and AlphaFold3-Server predictions, with the 10-run best TM-Scores displayed below. In several cases, AlphaFold3-Server did not produce ribbon-like structures but gave structures resembling globular proteins (Fig. 3), whereas, as was intended, RibbonFold consistently generated parallel-in-register, ribbon-like structures.

Ablation experiments were carried out to identify the contributions of various mechanisms to its performance (*SI Appendix*, Fig. S1), and those components in Fig. 1 are critical. *SI Appendix*, Fig. S1 provides a detailed breakdown of these ablations, demonstrating the diverse factors influencing RibbonFold’s accuracy. Removing ribbon constraints led to a 0.045 decrease in the best TM-score, while using the standard loss resulted in a more significant 0.065 reduction. Excluding MSA input or the initial structure also negatively impacted performance. Freezing parameters in AlphaFold2 modules did not yield noticeable improvements.

## Comparing the Polymorph Landscapes of A*β*_1−42_ with the Landscapes of A*β*_1−40_ and A*β*_12−42_

It is worthwhile to explore the generated structural ensembles for specific cases and relate the results to the amyloid energy landscapes.

**Fig. 4.**
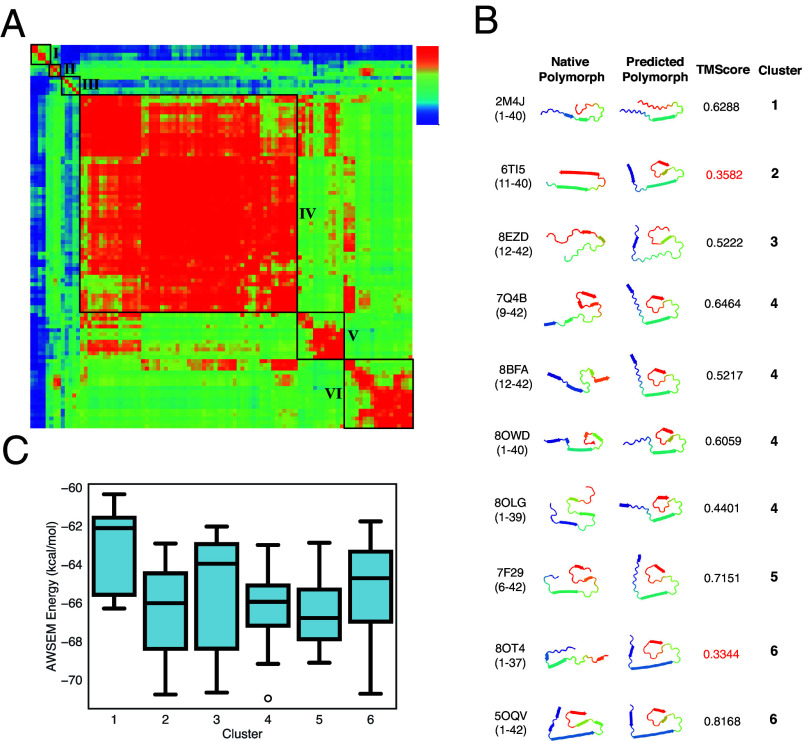
The polymorph landscape of the monomeric A*β*_1−42_ ribbon generated by RibbonFold. (*A*) Clustered polymorphs for 100 predictions of five A*β*_1−42_ peptides in a monomer protofilament, using mutual-Q as the metric for measuring structural similarity. One hundred predicted ribbon structures were hierarchically clustered and are shown in a heatmap on the *Left*. The identified clusters are enclosed in black squares on the heatmap, and the centroid structure from each cluster is shown and colored according to the sequence index from blue (N-terminal) to red (C-terminal) on the *Right*. (*B*) A representative polymorph from each predicted cluster is shown in a 2D ribbon form, and their corresponding native polymorph is also found and shown in accompany. (*C*) Binding energies of adding an additional monomer in different clusters computed using AWSEM. These binding energies are thus related to the thermodynamic equilibrium solubilities.

We first focus on the polymorph landscape of A*β*_1−42_ that was generated using RibbonFold to give 100 predicted structures. Clustering analysis, based on the mutual-Q similarity measure, on the 100 predicted structures allows us to visualize and quantify the similarities and differences between the predicted structures (Fig. 4*A*). We will identify each tight and significantly populated cluster as a “polymorph.” These polymorphs, along with their average energies, represent the polymorph landscape. For the A*β*_1−42_ peptide, five significantly populated clusters covering all of the predictions were obtained by this clustering analysis.

Fig. 4*B* provides a summary of the structural comparisons between the predicted polymorph cluster centroid structures (predicted polymorph) and the available experimentally determined A*β* fibril polymorphs (native polymorph). The figure also includes similar comparisons of the various experimental structures with each other in terms of TM-Score. Around 10 types of native polymorphs can be identified from all the experimentally solved A*β* related fibrils covering different lengths (Fig. 4*B*). It is worth noting that seven of the experimental polymorphs can find a good match among the computed polymorphs from RibbonFold with a TM-score above 0.5. These different polymorphs contain both “U”-shaped fibrils (in cluster 1) and “S”-shaped fibrils (in the other clusters). Furthermore, the experimental structures cluster into two dominant protofilamentary ribbon folds depending on whether or not residues 41 and 42 are present. Those constructs that include residues 41 and 42 (A*β*_12−42_ and A*β*_1−42_) have S-shaped ribbon folds and are ribbon dimers. Those constructs that lack residues 41 and 42 (A$\beta_{1-40}$ and A$\beta_{9-40}$) have U-shaped ribbon folds and form either ribbon trimers or ribbon dimers. Thus, assuming that the ribbon oligomer landscape selects from relatively stable ribbon monomer conformations, the experimental studies suggest that, grossly speaking, there are two dominant ribbon monomer folds of the A*β* sequence: U-shaped structures and S-shaped structures (Fig. 4). This dominance of two forms is also reflected by polymorph landscape of A$\beta_{1-40}$ (*SI Appendix*, Fig. S4), which contain more non-S-shaped fibrils.

To have an energetic picture of the different clustered polymorphs, we plot the AWSEM energies computed for each cluster. Structures in clusters 2, 3, 4, 5, and 6 with an S-shaped ribbon have the most favorable binding energy upon the addition of free monomers. While the U-shaped cluster 1 is much less favorable. according to the AWSEM energies.

## Polymorph Landscapes of Scrambled A*β*_1−42_ and A*β*_1−40_ Polypeptides

In the polymorph landscape of A*β*_1−42_ (Fig. 4), A$\beta_{1-40}$ (*SI Appendix*, Fig. S4) and A$\beta_{12-42}$ (*SI Appendix*, Fig. S5), the modest number of polymorphs predicted in annealing runs parallels the rather small number of structures that have been characterized in detail in the laboratory over the years. The existence of a dominant cluster and several minor clusters seems to suggest some degree of funneling to the landscape but with substantial ruggedness for the ribbon landscape for the natural sequence.

**Fig. 5.**
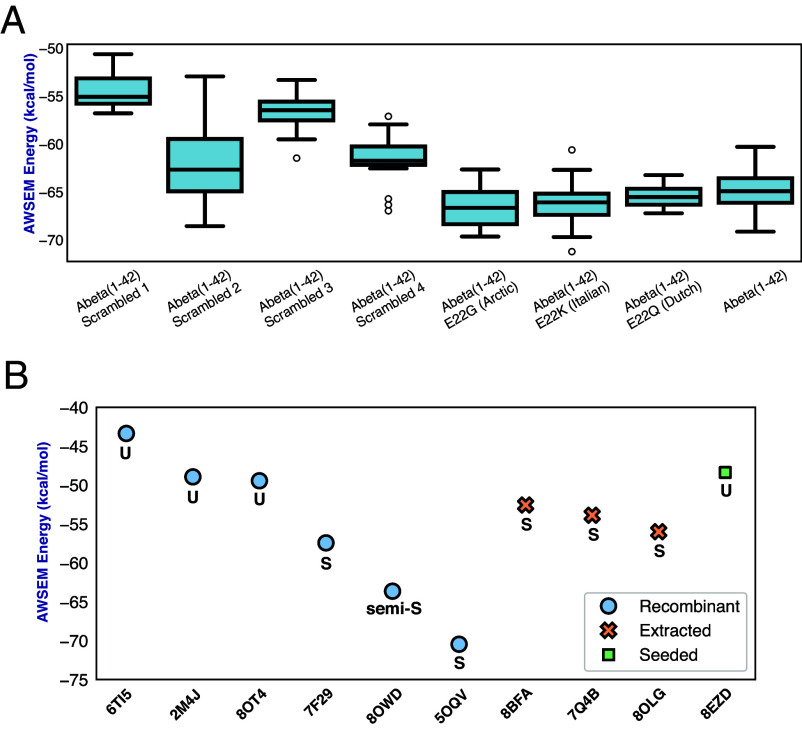
Comparing the elongation energetics of $A\beta_{1-42}$ from different sources and with scrambled sequences. (*A*) In total, 20 predicted ribbons for each of 4 scrambled sequences were generated, and the binding energy values of each predicted ribbon were computed from AWSEM to compare with the energetics from the ribbons predicted for WT A$\beta_{1-42}$ sequence and the E22G (Arctic), E22K (Italian), and E22Q (Dutch) mutants. The energetic distribution is illustrated as a boxplot for each ensemble. (*B*) In comparison, experimentally observed ribbons of $A\beta_{1-42}$ from different sources are also evaluated for their AWSEM energies. The colors indicate their corresponding experimental sources. In total, six ribbons from recombinant proteins, one ribbon from seeding experiments and three ribbons from direct patient samples were evaluated. The structures of these ribbons are shown in Fig. 4*B* and are generally classified as S-, U-, and semi-S-shaped ribbons. The shape of each structure is displayed below the markers.

It is likely that the paucity of structures generated in the polymorph funnel arises from a correlation with solubility. The energies then might give us information on this form of selection. We therefore also surveyed the polymorph landscapes for four scrambled A$\beta_{1-42}$ sequences (Fig. 5*A*). Although RibbonFold generated several clusters for those scrambled variants, the structures were significantly less stable energetically than those generated for natural $A\beta_{1-42}$, suggesting these sequences are more soluble than natural Aβ (Fig. 5*A*). Elongation energies of the scrambled sequences show they are at least 3.5 kcal/mol less stable than the wild type, meaning they would be at least 300 times more soluble than the wild type amyloids. These results together suggest that there may indeed be some selection on the basis of solubility explaining the polymorph funnel. Similar results were also observed for $A\beta_{1-40}$ (*SI Appendix*, Fig. S6). Some have suggested that amyloid fibrils may usefully sequester peptides that might otherwise form harmful soluble oligomers. Such a protection notion would suggest that there is perhaps a survival value for the organisms favoring facile aggregation but this is less clear. At the same time, predictions of some famous mutants were made and evaluated energetically. Both the Arctic (E22G) and Italian (E22K) mutants are significantly more stable than the wild type, while the predictions for the Dutch (E22Q) mutant are only slightly more stable than natural Aβ. The Italian and the Arctic mutants have been reported to aggregate faster, displaying a shortened, sometimes undetectable, lag phase (53, 54), consistent with their greater stability.

A$\beta_{1-42}$ fibrils with solved structures typically are made from recombinant proteins in vitro, or seeded in vitro from samples from patients’ brains or directly extracted from patients’ brains. When we examine the elongation energies of experimentally solved structures, we see that the elongation energies of S-shaped amyloids are more favorable than those of U-shaped polymorphs, indicating that the S-shaped polymorphs have a lower solubility (Fig. 5*B*). Different recombinant versions have been studied at various experimental concentrations, with both S and U-shaped polymorphs. All the three polymorphs extracted from patients show the more stable S-shaped format, suggesting that disease-causing polymorphs might be selected by their being more stable and thus less soluble.

## Amyloid Polymorphism of Tau_305−379_ Sequences

Moving on to larger polypeptides, we examined the predicted polymorph landscape of Tau_305−379_ segments. In the human brain, alternative splicing of the tau pre-mRNA results in six molecular isoforms of this protein. These six Tau isoforms differ from each other by containing either three (3R Taus) or four (4R Taus) microtubule binding repeats (R). The polymorph landscape of 3R-4R Tau_305−379_ is shown in Fig. 4 after 100 calculations using RibbonFold.

Similar to what was seen for *Aβ*, we were able to identify tight and significantly populated clusters corresponding to distinct polymorphs. These polymorphs, along with their average energies, can be taken as representing the polymorph landscape. For the Tau-3/4R peptide, five significantly populated clusters covering most of the predictions were obtained by clustering analysis, but some singletons were also observed after clustering analysis.

**Fig. 6.**
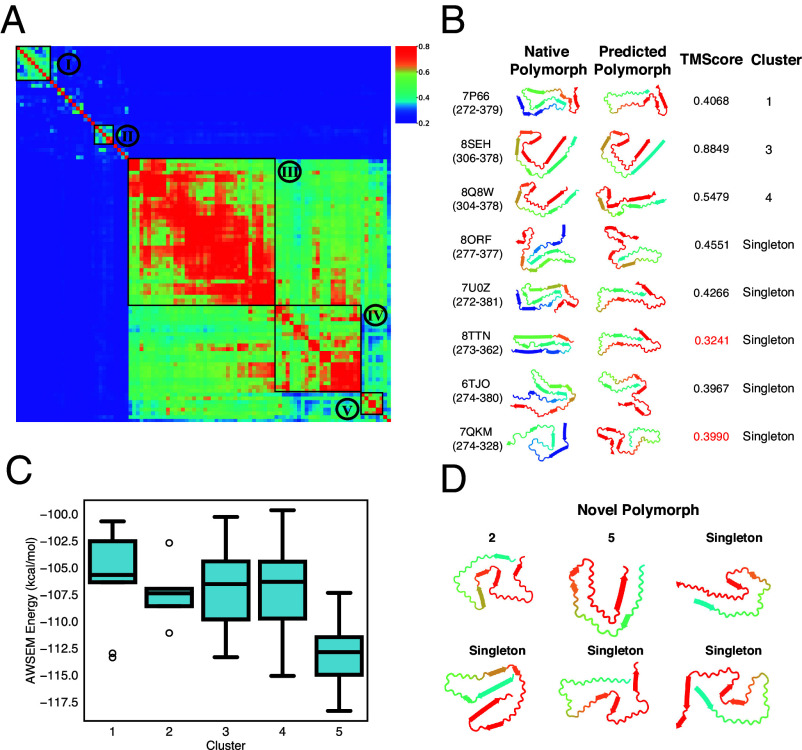
Polymorph landscape of the monomeric Tau_305−379_ ribbon predicted by RibbonFold. (*A*) Clustered polymorphs for 100 predictions of five Tau_305−379_ peptides in a monomer protofilament, using mutual-Q as the metric for measuring structural similarity. One hundred predicted ribbon structures were hierarchically clustered and are shown in a heatmap on the *Left*. The identified clusters are enclosed in black squares on the heatmap, and the centroid structure from each cluster is shown and colored according to the sequence index from blue (N-terminal) to red (C-terminal) on the *Right*. (*B*) The representative polymorph from each predicted cluster is shown in a 2D ribbon form, and their corresponding native polymorph is also found and shown in accompany. Some predicted polymorphs do not have a well-defined experimental hit and are shown as singleton polymorphs. (*C*) Binding energies of involving an additional monomer in different clusters computed using AWSEM. (*D*) Representative novel polymorphs identified from 100 predictions, where these polymorphs do not have corresponding experimental hits.

Fig. 6*B* provides a summary of structural comparisons between the predicted polymorphs and the known amyloid polymorphs. We also include similar comparisons of the different experimental structures with each other in terms of their TM-Scores. Around eight types of native polymorphs were identified from all the experimentally solved Tau related fibrils covering different lengths (Fig. 6*B*). It is worth noting that six of the polymorphs do find a good match from the computed polymorphs from RibbonFold with a TM-score above 0.4. Those different polymorphs contain both the common AD fold (cluster 1, 3, 5) (55) and PiD fold fibrils (cluster 4) (56). Besides these, we have also found that some generated polymorphs from RibbonFold that do not have good matches against known natural polymorphs, and must thus be classified as novel polymorphs. Considering the fact that the experimental studies have been limited yet in exploring the space of polymorphs for Tau, we believe that these novel polymorphs may will await experimental validations.

## Fibril Polymorphism of *α*-Synuclein_1−99_ Sequences

We also examined the polymorph landscape of *α*-Synuclein_1−99_ segments. Again, one can identify tight and significantly populated clusters corresponding to polymorphs. For the *α*-Synuclein peptide, six significantly populated clusters cover 95% of the predictions while some singletons were also observed after clustering analysis.

**Fig. 7.**
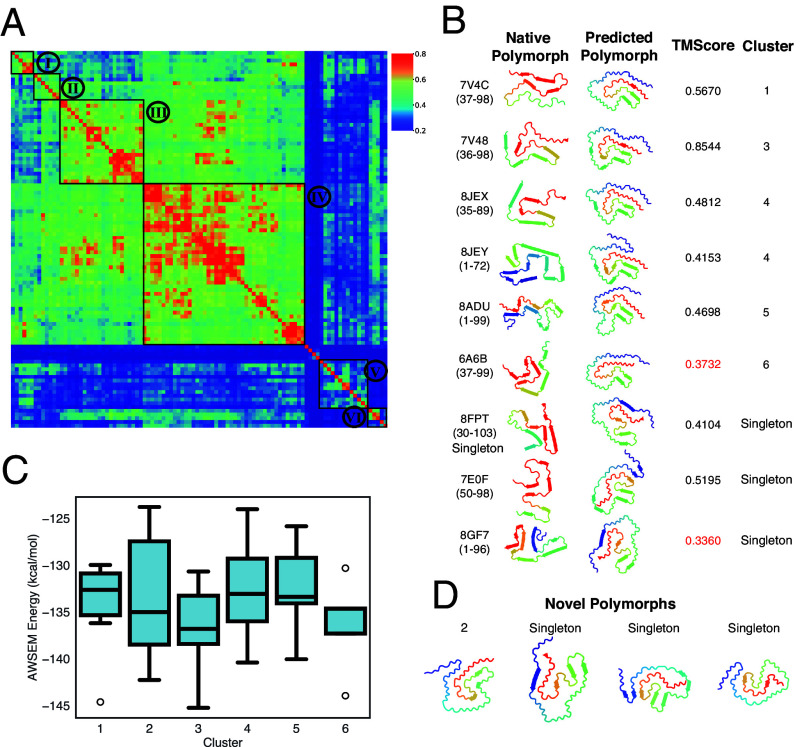
Polymorph landscape of the monomeric *α*-synuclein_1−99_ ribbon predicted by RibbonFold. (*A*) Clustered polymorphs for 100 predictions of five *α*-synuclein_1−99_ peptides in a monomer protofilament, using mutual-Q as the metric for measuring structural similarity. One hundred predicted ribbon structures were hierarchically clustered and are shown in a heatmap on the *Left*. The identified clusters are enclosed in black squares on the heatmap, and the centroid structure from each cluster is shown and colored according to the sequence index from blue (N-terminal) to red (C-terminal) on the *Right*. (*B*) The representative polymorph from each predicted cluster is shown in a 2D ribbon form, and their corresponding native polymorph is also found and shown in accompany. Some predicted polymorphs do not have a well-defined experimental hit and are shown as singleton polymorphs. (*C*) Binding energies of involving an additional monomer in different clusters computed using AWSEM. (*D*) Representative novel polymorphs identified from 100 predictions, where these polymorphs do not have corresponding experimental hits.

Fig. 7*B* provides a summary of structural comparisons between the predicted polymorphs and the presently observed polymorphs. We also include comparisons of the different experimental structures with each other in terms of TM-Score. Nine types of native polymorphs are then identified from all the experimentally solved *α*-Synuclein related fibrils covering different lengths (Fig. 7*B*). We observe that 7 of the polymorphs do find a good match from the computed polymorphs from RibbonFold with a TM-score above 0.4. Clusters 1, 2, 3, 4, and 5 all exhibit the conserved amyloid core formed by *α*-Synuclein_45−90_, a core that has been frequently observed in known solved structures. Again, some generated polymorphs from RibbonFold do not find a good match against presently known polymorphs and are thus classified as novel polymorphs.

## Discussion

### Glassy Features of the Amyloid Folding Landscape.

**Fig. 8.**
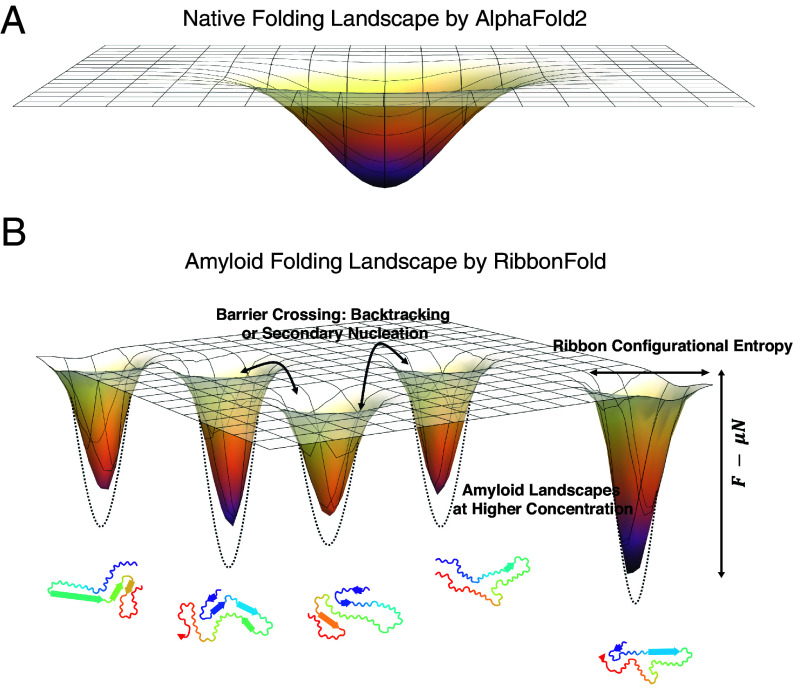
A schematic diagram of the amyloid folding landscape in comparison with globular protein landscapes. (*A*) The globular energy landscape is usually funneled toward the native state. While there are rugged local minima and barriers, the general outcome is to easily reach the native state at low temperature. (*B*) Explorations of amyloid folding landscape with RibbonFold show that there might be a limited number of preferred states in the amyloid state, but the transition between the states would be kinetically impossible without dissolution. Different environmental factors like pH, salt, binding partners, etc., will affect the polymorph landscape. The depth represents the grand canonical free energy, F−Nμ, as a function of oligomer size and the concentration of free monomers. At a higher concentration, the potential wells would be further deepened (dashed sketches).

The current version of RibbonFold generates around 5 to 10 polymorphs for each peptide. Rather often the amyloid folding landscape has been pictured as having high barriers between basins. In monomeric protein folding to a native structure, the depth of the funnel corresponds to the standard free energy of individual configurations which can directly interconvert. The aggregation landscape, in contrast, depends on concentration. The corresponding thermodynamic potential is the free energy minus the (concentration dependent) chemical potential times the number of monomer units in an aggregate in a specific polymorph (Fig. 8). Aggregates interconvert by growing or shrinking thereby changing N typically but could at fixed N simply reconfigure as in the backtracking process (28). We see that the ribbon constraints imply the thermodynamic potential landscape for ribbon structures alone generally does not properly picture direct interconversion of polymorphs but reflects solubility and thus the possibility of interconverting through secondary nucleation or kinetic replacement (57).

### Origins of Small Multiplicity of Amyloid Polymorphs.

The RibbonFold algorithm suggests that these well-known amyloid-forming sequences possess a limited number of plausible polymorphs on their “solubility” landscape (Fig. 8). To examine the extent to which sequence selection plays a role in shaping this polymorph landscape, we also examined the landscapes of scrambled versions of Tau_305−379_ and *α*-synuclein_1−99_. We found that the scrambled versions of both Tau and *α*-synuclein are not very much less stable energetically than the wild types, but nevertheless the wild types do lead still to the most stable ribbon structures.

The polymorph landscapes were also obtained for prion-like domains in functional prions like Orb2A (*SI Appendix*, Fig. S10), apCPEB (*SI Appendix*, Fig. S11), sup35−*NM* (*SI Appendix*, Fig. S12), and Ure2p (*SI Appendix*, Fig. S13). Generally, the clusters are smaller, and the ribbons exhibit a greater degree of flexibility. These proteins are thought to retain physiological functions even in their amyloid form, suggesting that the diversity of polymorphs may arise from functional selection processes (18, 58). In comparison with a single-funnel landscape of globular proteins which guides folding to a kinetically accessible free energy minimum, this multifunnel amyloid landscape has alternative kinetic traps (i.e., addition of monomers). This multifunnel nature is the combinatorial outcome of both thermodynamics and kinetics. The current RibbonFold predictions are not intended to provide a realistic kinetic description of the formation of amyloids. Once stable amyloid polymorphs have been predicted using RibbonFold, however, we can use importance sampling without having the ribbon constraints in place to investigate both the solubility and the structural mechanism of formation of the predicted polymorphs, as we have done previously using experimentally determined fiber structures as input (37).

### RibbonFold Provides a Useful Framework for Structurally Characterizing Amyloid Polymorphism Landscapes.

While many proteins are known to form amyloid fibrils above a critical concentration that depends on both the sequence of the aggregating polypeptide and on the solvent conditions, only a handful of amyloid fiber structures, however, have been determined by experimentalists in detail. Detailed determination of protein fibril structures has proved, in many ways, substantially more difficult than the experimental determination of globular protein structures. While Alphafold2/3 generates high-quality structural models for globular proteins from sequence, corresponding theoretical and machine learning tools in the area of amyloids have mostly been limited to identifying those short peptides that seem most responsible for driving amyloid formation (25, 59–62). By viewing the problem of predicting the structures of stable amyloid polymorphs as a two-dimensional protein-ribbon folding problem, RibbonFold provides a practical algorithm that can, in principle, predict the most stable overall amyloid folds of any sequence.

### Limitations and Extensions of the Current Ribbon Folding Scheme.

While detailed experimental characterization of the structures of amyloid folds is by itself challenging, directly probing the energetics of amyloid folds is more challenging still. We caution that ribbon predictions do not provide a realistic kinetic description of the mechanisms of formation of amyloid fibers. Other tools are needed to explicitly study how the predicted polymorphs would arise and evolve under different conditions via primary nucleation, secondary nucleation, and elongation. We emphasize however that coarse-grained simulations are already proving quantitatively useful on this score (9, 31, 37).

It is worth noting that the current version of RibbonFold achieves a best TM-score of around 0.5, which remains modest for structure prediction. This performance can be attributed to two primary factors: the limited size of the database and the highly polymorphic nature of most fibrils. Further improvements are underway.

There are several limitations of the RibbonFold approach presented here that are worth noting. The current version of the algorithm assumes that the most stable amyloid polymorphs will be based on parallel in-register *β*-sheets. This assumption is consistent with the majority of amyloid fibril structures that have been determined so far by experiment in detail. Nevertheless crystal structures of amyloid fibers with parallel out-of-register *β*-strands have been observed, but these are for very short peptides (≈6 residues) (63, 64). The out-of-register parallel *β*-sheet does not appear to be favorable for the longer peptides that form amyloid fibers. Tycko and coworkers have presented a structure of a metastable antiparallel A*β* fibril structure formed by A*β* molecules containing the disease-causing “Iowa” mutation (40). This antiparallel form, however, eventually converts to a parallel fibril structure which therefore must be lower in free energy. Another more subtle limitation is associated with the existence of cofactors and salt ions, which are critical in modulating fibril architecture. For instance, cryo-EM studies of Tau reveal that distinct fibril folds correlate with varying densities of bound cofactors (65). Moreover, Tau fibrils derived from brain samples display significant structural differences compared to those assembled in vitro; notably, altering salt conditions in vitro can induce the formation of Tau folds that more closely resemble their in vivo counterparts (66).

In addition to the assumption of parallelism, RibbonFold at this time is not able to handle higher-order amyloid fibrils composed of multiple amyloid protofilaments. It would be useful to fully exploit the capability by incorporating oligomeric ribbons in the training scheme. Experimentally determined structures of mature A*β* and IAPP fibrils typically contain more than one protofilamentary stack, indicating that the lateral association of protofilaments probably contributes to the stability of these structures (67). Those lateral associations can be very weak but critical as observed from solved structures (23, 67). There are around 100 solved amyloid structures with multimeric ribbons, i.e., around 20 amyloid structures of Tau contain more than one protofilaments. Training that includes these multimeric ribbons would potentially benefit the current RibbonFold method through providing more meaningful interactions that can be learned.

## Materials and Methods

### Curation of Structures of Amyloid Fibrils.

Our raw data used in training and testing were obtained from Amyloid Atlas 2024. For structures containing multimeric ribbons, each ribbon was isolated, yielding a total of 822 individual ribbons. Structures in which the interribbon contacts dominate were excluded, because the current scheme is to study the folding of monomeric ribbons. Following this construction, each ribbon was saved containing five monomer chains: ribbons with more than five chains in the database were truncated to five, while those cataloged with fewer chains were extended periodically. Next, we performed sequence-based clustering on these monomeric ribbons, dividing them into 42 distinct sequence clusters. Structure-based clustering using mutual-Q was then conducted within each sequence cluster, resulting in 220 structure clusters in total (Fig. 2*A*). During the training process, each training epoch involved iterating over these predefined structure clusters, with a random structure being selected from each cluster to ensure that all the 42 clusters are sampled across during training.

### Encoding Parallel-in-Register Constraints into AlphaFold2 for the Prediction of Amyloid Fibrils.

Almost all explicitly solved amyloid fibril structures have a parallel in-register arrangement of the hydrogen bonds, with each monomer approximately spanning across one layer in its fibril form in two dimensions, like a ribbon. Parallel-in-register constraints are encoded into AlphaFold2 through the template module. We designed a customized template feature based on our prior knowledge of amyloid fibril structures, specifically encoding their interchain distance and parallel orientation. In this custom template pair matrix, entries with the same residue index but different chain index are utilized and the rest of the entries are masked. For each entry in use, the distance between corresponding residues is encoded: this is taken to be approximately 4.85$\overset{˚}{A}$ for adjacent chains, and 4.85$\overset{˚}{A}$ × k for chain pairs separated by k chains. This encoding is carried out by discretizing the distance values into one-hot vectors, similar to AlphaFold2’s approach. Additionally, a universal random unit vector is concatenated to each of these entries to signify the parallel-in-register characteristic of the chains.

### A Polymorph Loss Function to Address the Multiplicity of Polymorphisms.

Given that a single amyloid sequence may correspond to multiple polymorphic structures, a polymorph loss function recognizing this multiplicity was implemented during training. This function involved computing loss across multiple predictions and multiple target structures and averaging for each of the samples. Specifically, for each input sequence, M forward passes are performed to produce M predicted structures (*P*_*i*_), which are then compared with N-known polymorphic target structures (*T*_*j*_) that are associated with the sequence. The loss between *P*_*i*_ and *T*_*j*_ is defined as a combination of FAPE loss, distogram loss, and conflict loss obtained from AlphaFold2. These target structures are selected from the dataset based on sequence similarity while ensuring structural diversity based on their computed mutual-Q, with up to six distinct target structures for each sequence. For each target, the minimum loss across the M predictions is selected, and these N minimum losses are averaged to obtain the final polymorph loss for the set. To demonstrate that RibbonFold can generate a range of polymorphic structures, we sampled 100 times and conducted clustering analysis for each test sequence. The clustering analysis included calculating pairwise Q-scores and comparing TM-scores against native polymorphic structures (Fig. 3).

[1] $$L_{Polymorph}=\frac{1}{N}\cdot \sum_{j=1}^{N}\underset{1⩽i⩽M}{\min}\left(L_{ij}\right),$$

[2] $$L_{ij}=L_{FAPE}(P_{i},T_{j})+0.5\;\cdot \;L_{dist}(P_{i},T_{j})+0.01\;\cdot \;L_{conflict}(P_{i},T_{j}).$$

### Sampling RibbonFold.

To bring greater diversity into the model predictions, we used random MSA subclustering (47). For each sampling instance, a single MSA row is randomly selected from the MSA cluster centers derived from AlphaFold2, while the extra MSAs remain unchanged. This is inspired by the observation that different MSA clusters can result in different structure conformations, and shallow MSA can increase sample diversity. We also apply MSA column dropout to both the selected MSA center and all extra MSAs to further enhance diversity in the input representation. We see that the MSA’s function to some extent as stochastic noise in the generation of predicted structures.

### Clustering Analysis of Predicted Ribbon Structures.

A hierarchical algorithm, with a “centroid” linkage scheme, was used to cluster the predicted ribbon structures. We obtained clusters by using the set of mutual-Q order parameters to build the linkage matrix (68). *Q* as defined in Eq. **1** is symmetric. The mutual-Q value for a pair of structures is obtained by assuming that one of the structures is a basic “native” or local structure (has pair distances $r_{\mathrm{ij}}^{N}$) and computing the *Q* of the other structure using this local structure as a reference. Clusters of structures are identified as being those sets of structures that have an average mutual-Q value within the cluster greater than 0.4. The centroid structure of each cluster is visualized in the accompanying figures. The mean value of the mutual-Q inside each cluster roughly describes the tightness of the cluster.

[3] $$\begin{array}{cc} Q & =\frac{2}{\left(N-2)(N-3\right)}\sum_{j>i+2}exp\left[-{\left(r_{\mathrm{ij}}-r_{\mathrm{ij}}^{N}\right)}^{2}/2\sigma_{\mathrm{ij}}^{2}\right]. \end{array}$$

### Training Procedures.

Our training procedure basically follows the one used for AlphaFold2. For each training item, we identify and incorporate its known polymorphic structures. Those structures along with the current structure are used for calculating polymorph loss. We apply symmetric residue cropping, ensuring consistent cropping across all chains, with a maximum cropping length of 384 residues. The model was trained using the AlphaFold2.3.1 checkpoint (Model 2) on a cluster of 24 NVIDIA A100 GPUs over approximately 3 d.

## Supplementary Material

Appendix 01 (PDF)

## Data Availability

All predicted structures presented are available on Zenodo at https://zenodo.org/records/15123274 (69). Code for running RibbonFold has been released on GitHub, free for academic, personal, and commercial use at https://github.com/Mingchenchen/RibbonFold (70). All study data are included in the article and/or *SI Appendix*.
