# Supplementary material for: Generating the polymorph landscapes of amyloid fibrils using AI: RibbonFold
Source: Proc Natl Acad Sci U S A. 2025 Apr 15;122(16):e2501321122. doi: 10.1073/pnas.2501321122 (PMC12037047; doi:10.1073/pnas.2501321122)
Supplement: Supplementary file 1 — Appendix 01 (PDF) [file pnas.2501321122.app.pdf]

Generating the Polymorph Landscapes of Amyloid Fibrils Using Artificial Intelligence: RibbonFold

Liangyue Guo[1,4], Qilin Yu[1,4], Di Wang[1], Xiaoyu Wu[1], Peter G. Wolynes[2,3], and Mingchen Chen*[1]

[1]Changping Laboratory, Beijing, China
[2]Center for Theoretical Biological Physics, Rice University, Houston, TX
[3]Department of Chemistry, Rice University, Houston, TX
[4]These authors contributed equally to this work
*Email: mingchenchen@cpl.ac.cn

correspondence to: mingchenchen@cpl.ac.cn

Contents

S1 Algorithm details **S2**

S2 Supplementary results on RibbonFold **S3**
S2.1 Detailed results of RibbonFold v.s. AF3-Server on two test sets S3
S2.2 Ablation studies on RibbonFold S5
S2.3 Comparing the effects of using different ribbon sizes during inference on the first test set S7
S2.4 Correlation between plDDT and TM scores S8

S3 Supplementary Results **S9**
S3.1 Fibril Polymorphism of Amyloid-β_{12-42} ribbon and Amyloid-β_{1-40} computed by RibbonFold. . S9
S3.2 Fibril polymorphism of scrambled Amyloid-β_{1-40} Polypeptides. S11
S3.3 Fibril polymorphism of scrambled Tau Polypeptides. S12
S3.4 Fibril polymorphism of scrambled α-Synuclein Polypeptides. S13
S3.5 Fibril polymorphism of TDP-43 Polypeptides. S14
S3.6 Polymorph landscape of Orb2, apCPEB-Q, Sup35 and Ure2p computed by RibbonFold S15
S3.7 Experimental correspondence of predictions for sup35$-NM$ S19
S3.8 Representative structures from polyQ peptides of different lengths computed by RibbonFold . . S20

S4 Polymorph landscapes computed from various proteins by AlphaFold3-Server **S22**

S1 Algorithm details

Algorithm 1 MakeParallelConstraintsTemplate

Require: $\boldsymbol{r}$: residue index, $\boldsymbol{c}$: chain index, $\boldsymbol{bins}$: vector for discretizing distance values

$\boldsymbol{a} \leftarrow \text{random}(3)$
$\boldsymbol{a} \leftarrow \frac{\boldsymbol{a}}{\|\boldsymbol{a}\|}$
for $i = 1$ **to** n **do**
    for $j = 1$ **to** n **do**
        if $\boldsymbol{r}_i = \boldsymbol{r}_j$ **and** $\boldsymbol{c}_i \neq \boldsymbol{c}_j$ **then**
            $d \leftarrow 4.85 \times |\boldsymbol{c}_i - \boldsymbol{c}_j|$
            $\boldsymbol{d} \leftarrow \text{one\_hot}\left(\sum_i (d > \boldsymbol{bins}[i])\right)$
            $\boldsymbol{a} \leftarrow \boldsymbol{a} \times (\boldsymbol{c}_i > \boldsymbol{c}_j)$
            $\boldsymbol{Z}_{ij} \leftarrow \text{concat}(\boldsymbol{d}, \boldsymbol{a})$
        end if
    end for
    $\boldsymbol{Z}_{ij} \leftarrow \text{Linear}(\boldsymbol{Z}_{ij})$
end for
return $\boldsymbol{Z}_{ij}$

Algorithm 2 PolymorphLoss

Require: s: input sequence, gt_struc: ground truth structure

$\text{poly\_strucs} \leftarrow \{struc \mid seq\_cluster[struc] = seq\_cluster[gt\_struc] \wedge structure\_cluster[struc] \neq structure\_cluster[gt\_struc]\}$
$\text{target\_strucs} \leftarrow \text{random.sample}(\text{poly\_strucs}, 5) \cup \{gt\_struc\}$
for $i = 1$ **to** M **do**
    $p_i \leftarrow \text{model}(s)$
    for $j = 1$ **to** $\text{len}(\text{target\_strucs})$ **do**
        $q_j \leftarrow \text{target\_strucs}[j]$
        $L_{ij} \leftarrow \text{Loss}(p_i, q_j)$
    end for
    $L_{\text{poly}} \leftarrow \frac{\sum_j \min_i (L_{ij})}{\text{len}(\text{target\_strucs})}$
end for
return L_{poly}

S2 Supplementary results on RibbonFold

S2.1 Detailed results of RibbonFold v.s. AF3-Server on two test sets

Table S1: Summary of TM-Scores On Testset

**Ribbon ID**	**Best TM-Score RibbonFold**	**Mean TM-Score RibbonFold**	**Best TM-Score AlphaFold3**	**Mean TM-Score AlphaFold3**
6cu8	0.4624	0.3702	**0.7876**	0.5582
6ic3	**0.4445**	0.3129	0.1551	0.1367
6qjh	0.6043	0.3696	**0.6842**	0.524
6wqk	**0.4743**	0.3166	0.3239	0.3054
7py2	**0.5093**	0.3785	0.3571	0.2672
7q3u	**0.4471**	0.3584	0.3155	0.2775
7vqq	0.3233	0.2754	**0.3387**	0.3077
7zir	0.3551	0.2758	**0.3864**	0.3406
8g54	**0.648**	0.5214	0.3104	0.2816
8olq	**0.5179**	0.377	0.3804	0.3584
8ons	0.2694	0.2413	**0.3477**	0.2882
8q8f	**0.533**9	0.3708	0.3661	0.304
8q96	**0.5015**	0.3934	0.4036	0.3624
8spa	**0.5028**	0.319	0.4328	0.3637
8ttn	**0.785**	0.4371	0.4624	0.3714

Table S2: Summary of TM-Scores On New Testset

**Ribbon ID**	**Best TM-Score RibbonFold**	**Mean TM-Score RibbonFold**	**Best TM-Score AlphaFold3**	**Mean TM-Score AlphaFold3**
9c1u	**0.5995**	0.4322	0.5086	0.3697
9jst	0.4223	0.3636	**0.4345**	0.3156
9jsu	0.4478	0.3576	**0.4732**	0.3494
9jsv	**0.5460**	0.4141	0.4732	0.3500

Table S3: Summary of LDDT & Chi1 & Chi2 accuracy On Testset

**Ribbon ID**	**Best LDDT RibbonFold**	**Best Chi1 accuracy RibbonFold**	**Best Chi2 accuracy RibbonFold**	**Best LDDT AlphaFold3**	**Best Chi1 accuracy AlphaFold3**	**Best Chi2 accuracy AlphaFold3**
6cu8	**0.2936**	0.7463	1.0000	0.2385	0.8293	0.9756
6ic3	**0.2924**	0.6923	0.7670	0.0911	0.5538	0.7956
6qjh	**0.3393**	0.8237	0.8847	0.2761	0.8136	0.8881
6wqk	**0.2623**	0.8070	0.7754	0.1425	0.7368	0.7228
7py2	**0.2417**	0.7797	0.8962	0.2187	0.7722	0.8734
7q3u	0.1907	0.7746	0.8732	**0.1995**	0.7183	0.8451
7vqq	**0.1803**	0.6242	0.9055	0.1675	0.6176	0.8901
7zir	0.2604	0.6392	0.7059	**0.2768**	0.7059	0.7059
8g54	0.1258	0.5077	0.7923	**0.1609**	0.5769	0.8462
8olq	0.2806	0.7750	0.7812	**0.2896**	0.7812	0.8125
8ons	**0.2324**	0.6215	0.8129	0.1528	0.6667	0.8387
8q8f	**0.4251**	0.7789	0.8816	0.2712	0.7684	0.8816
8q96	**0.1780**	0.7143	0.8643	0.1531	0.7321	0.8929
8spa	0.2269	0.6163	0.7673	**0.2600**	0.5306	0.7347
8ttn	**0.3799**	0.6422	0.8533	0.1875	0.7289	0.8867

Table S4: Summary of LDDT & Chi1 & Chi2 accuracy On New Testset

**Ribbon ID**	**Best LDDT RibbonFold**	**Best Chi1 accuracy RibbonFold**	**Best Chi2 accuracy RibbonFold**	**Best LDDT AlphaFold3**	**Best Chi1 accuracy AlphaFold3**	**Best Chi2 accuracy AlphaFold3**
9c1u	0.3399	0.6154	0.6615	**0.4858**	0.5769	0.7846
9jst	0.2416	0.6343	0.8171	**0.2434**	0.8286	0.8514
9jsu	0.2253	0.7394	0.8424	**0.2254**	0.7758	0.8727
9jsv	**0.2760**	0.7471	0.7706	0.2674	0.6412	0.8588

S2.2 Ablation studies on RibbonFold

To understand how RibbonFold predicts protein structure, we conducted ablation studies on RibbonFold's components to identify the contributions of various mechanisms to its performance. We performed six experiments: (1) No Ribbon Constraints: The template module was entirely disabled; (2)Standard Structure Loss: The standard AlphaFold2 structure loss was used; (3) No MSA Input: Multiple sequence alignments were not used; (4) No Initial Structure: Predictions were made from scratch, without an initial ribbon configuration; (5) Fixed Evoformer: Evoformer block parameters were frozen; and (6)Fixed Structure Module: Structure module parameters were frozen.

Figure S1 provides a detailed breakdown of these ablations, demonstrating the diverse factors influencing RibbonFold's accuracy. Removing ribbon constraints led to a 0.045 decrease in the best TM-score, while using the standard loss resulted in a more significant 0.065 reduction. Excluding MSA input or the initial structure also negatively impacted performance. Freezing parameters in AlphaFold2 modules did not yield noticeable improvements.

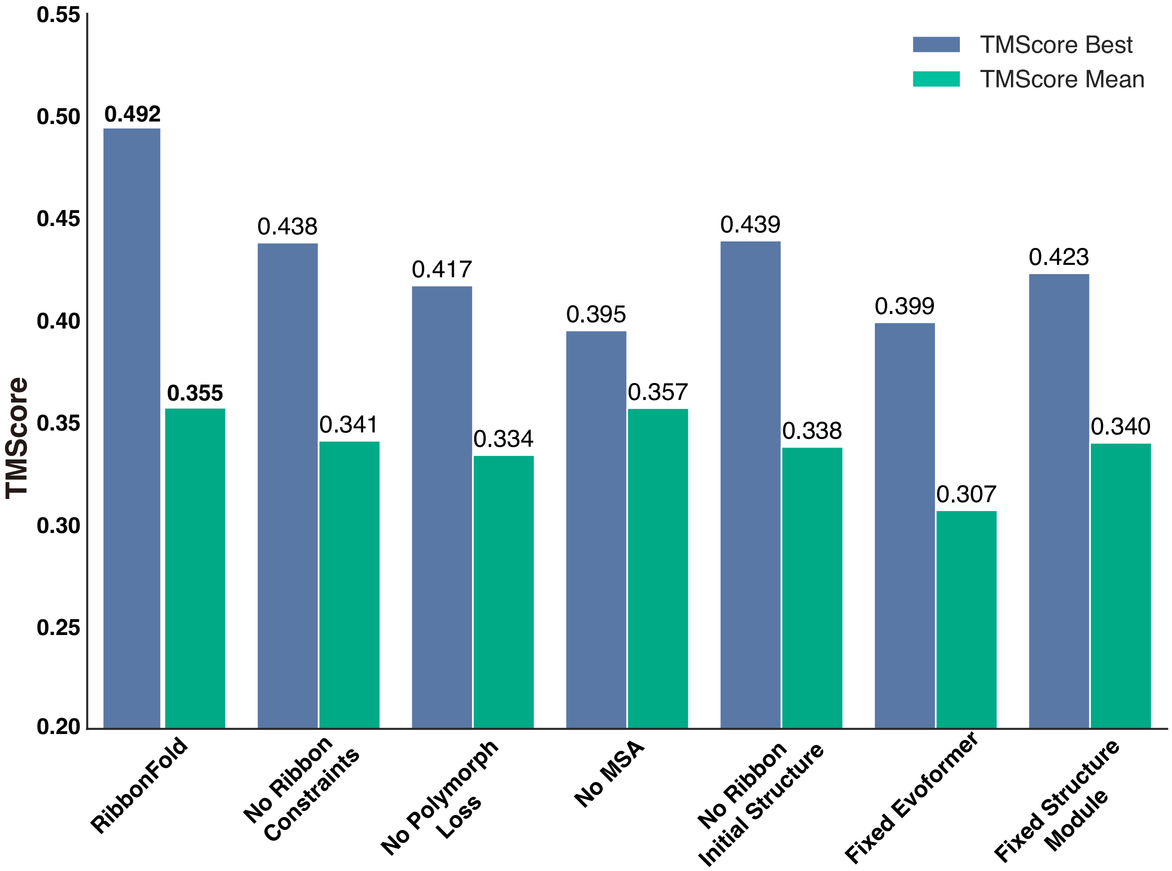

Figure S1: Ablation results on the first test set of 15 targets. For each target we carried out 10 predictions, and the performance of RibbonFold and its ablated versions are shown.

S2.3 Comparing the effects of using different ribbon sizes during inference on the first test set

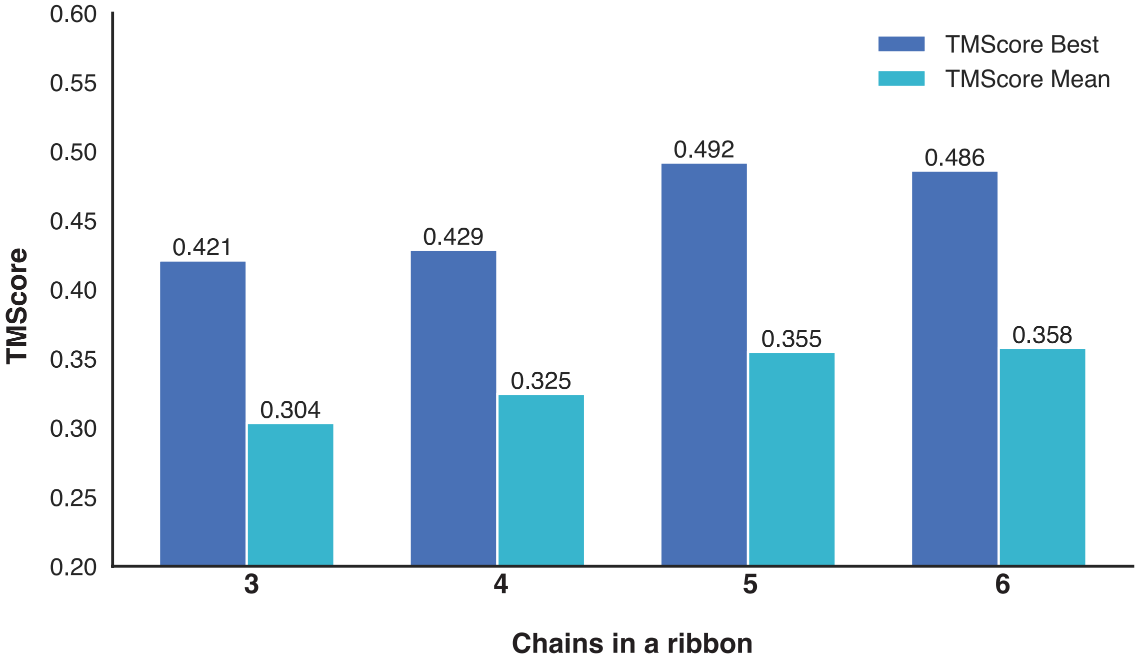

Figure S2: Comparison of performance of RibbonFold on the first test set when including different numbers of chains in a monomeric ribbon.

S2.4 Correlation between plDDT and TM scores

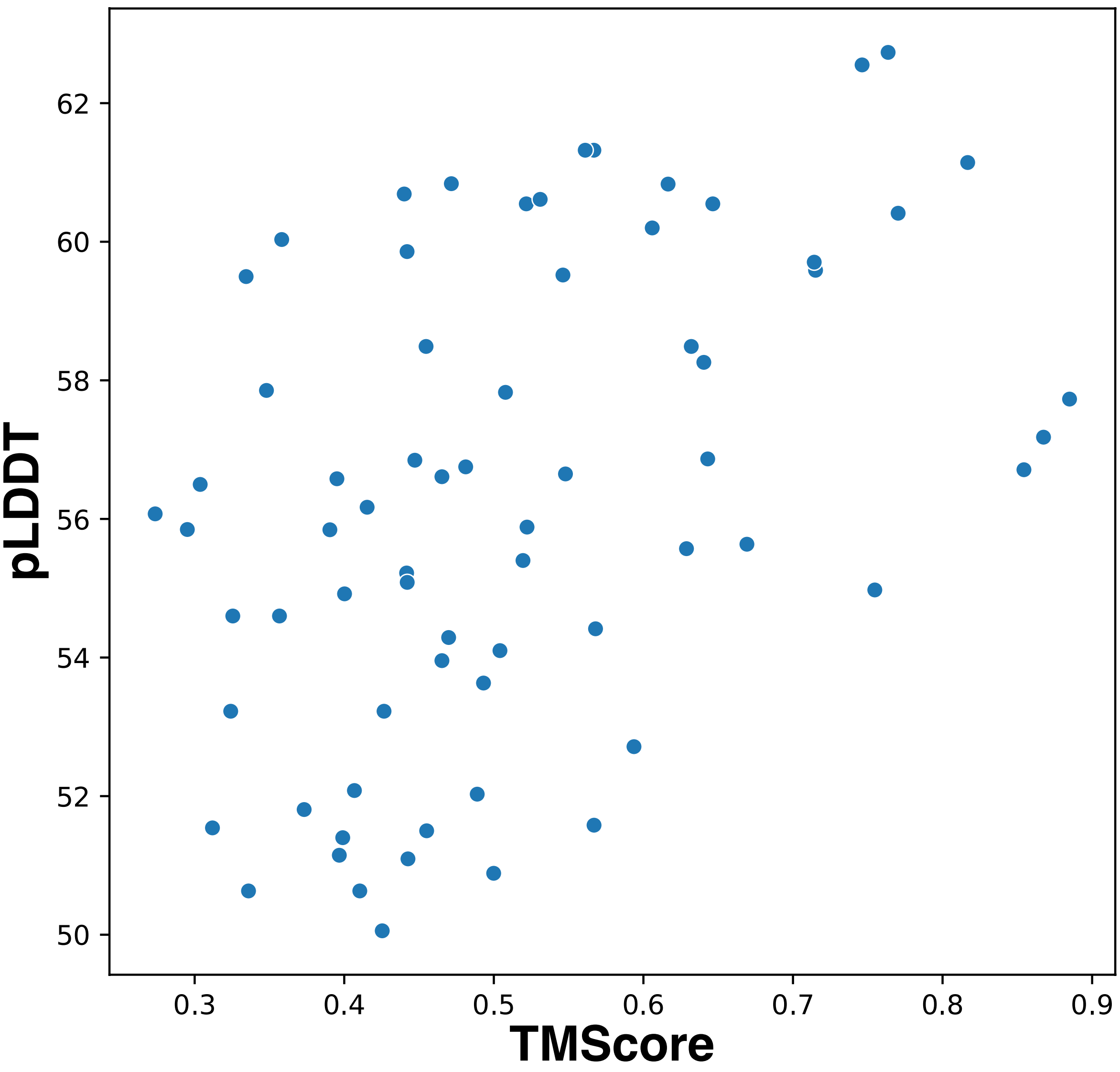

Figure S3: Correlation of plddt and TM scores. Because the dataset is limited, the ribbons with experimentally observed polymorphs were included. For each predicted structure, the best matched polymorph was found and the corresponding TM score was plotted. They in general have a Pearson correlation of 0.45.

S3 Supplementary Results

S3.1 Fibril Polymorphism of Amyloid-β_{12-42} ribbon and Amyloid-β_{1-40} computed by RibbonFold.

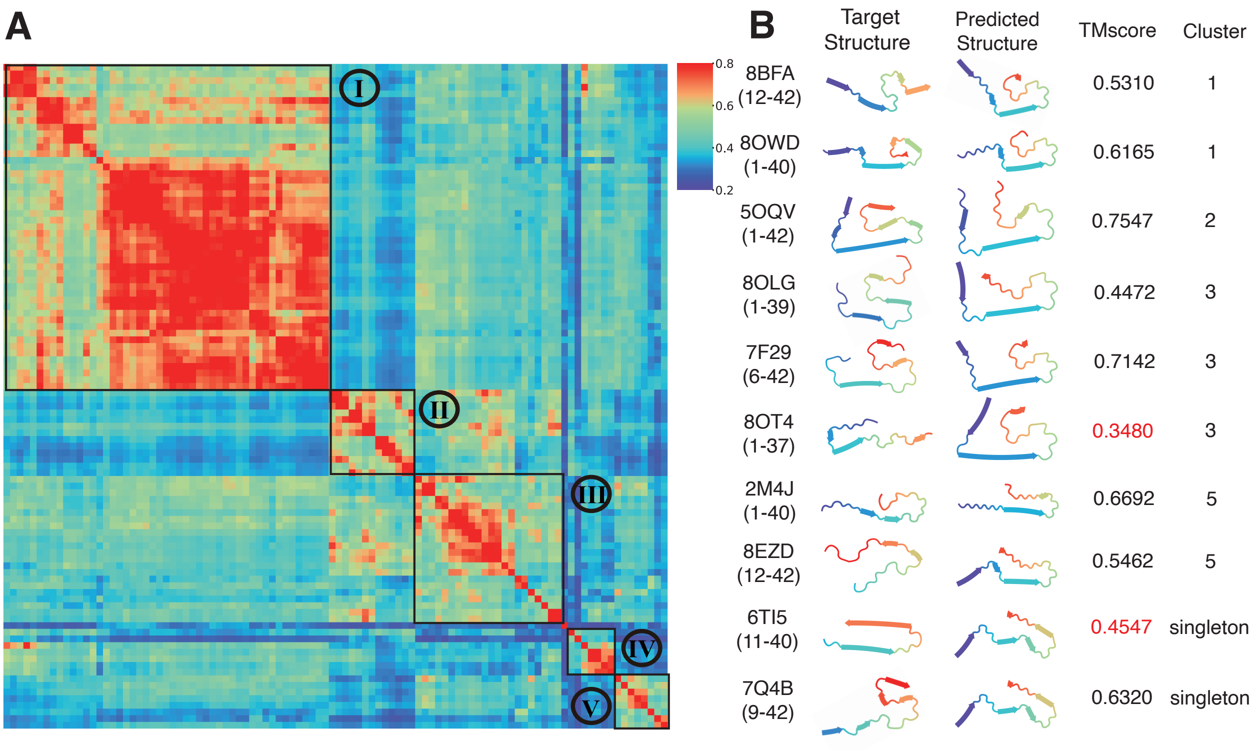

Figure S4: Polymorph landscape of the monomeric Aβ_{1-40} ribbon predicted by RibbonFold. A: Clustered polymorphs for 100 predictions of five Aβ_{1-40} peptides in a monomer protofilament, using mutual-Q as the metric for measuring structural similarity. One hundred predicted ribbon structures were hierarchically clustered and are shown in a heatmap on the left. The identified clusters are enclosed in black squares on the heatmap, and the centroid structure from each cluster is shown and colored according to the sequence index from blue (N-terminal) to red (C-terminal) on the right. B: The representative polymorph from each predicted cluster is shown in a 2D ribbon form, and their corresponding native polymorph is also found and shown in accompany. Some predicted polymorphs don't have a well-defined experimental hit, and are shown as singleton polymorphs.

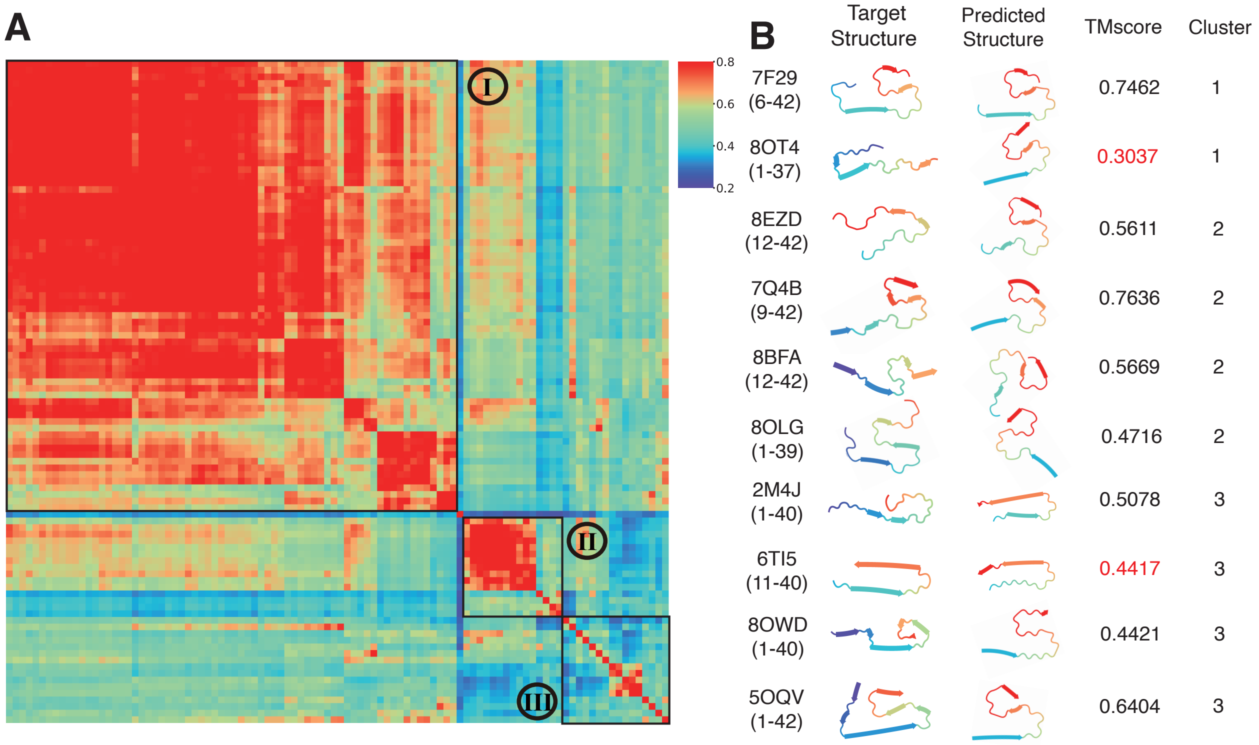

Figure S5: Polymorph landscape of the monomeric Aβ_{12-42} ribbon predicted by RibbonFold. A: Clustered polymorphs for 100 predictions of five Aβ_{12-42} peptides in a monomer protofilament, using mutual-Q as the metric for measuring structural similarity. One hundred predicted ribbon structures were hierarchically clustered and are shown in a heatmap on the left. The identified clusters are enclosed in black squares on the heatmap, and the centroid structure from each cluster is shown and colored according to the sequence index from blue (N-terminal) to red (C-terminal) on the right. B: The representative polymorph from each predicted cluster is shown in a 2D ribbon form, and their corresponding native polymorph is also found and shown in accompany. Some predicted polymorphs don't have a well-defined experimental hit, and are shown as singleton polymorphs.

S3.2 Fibril polymorphism of scrambled Amyloid-β_{1-40} Polypeptides.

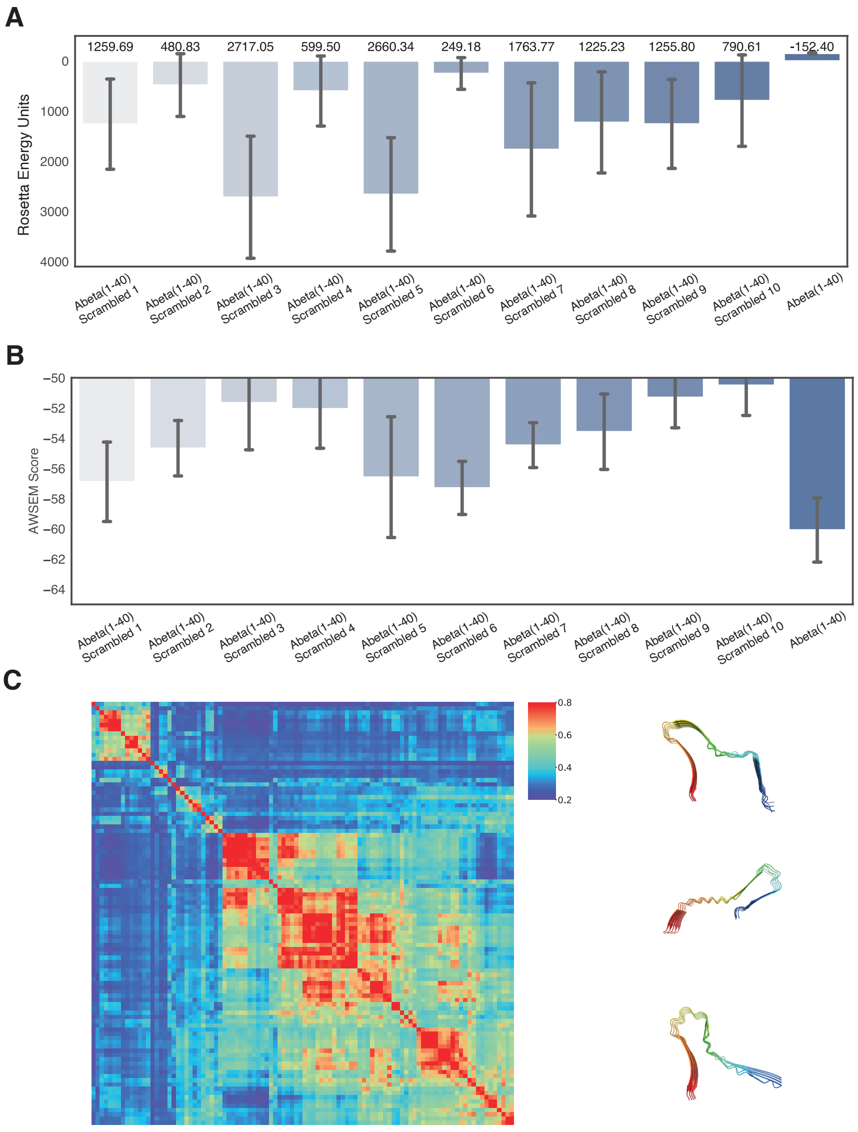

Figure S6: Polymorph landscape of a ribbon from an exemplar scrambled Aβ_{1-40} sequence predicted by RibbonFold. A: In total, 10 scrambled sequences were predicted, and the potential energy values of each ribbon was computed from Rosetta to compare with the energetics from WT Aβ_{1-40}. B: In total, 10 scrambled sequences were predicted, and the potential energy values of each ribbon was computed from AWSEM to compare with the energetics from WT Aβ_{1-42}. C: Clustered polymorphs for 100 predictions of an exemplar scrambled Aβ_{1-40} peptides in a monomer protofilament, using mutual-Q as the metric for measuring structural similarity. One hundred predicted ribbon structures were hierarchically clustered and are shown in a heatmap on the left. Three representative structures are shown and colored according to the sequence index from blue (N-terminal) to red (C-terminal) on the right.

S3.3 Fibril polymorphism of scrambled Tau Polypeptides.

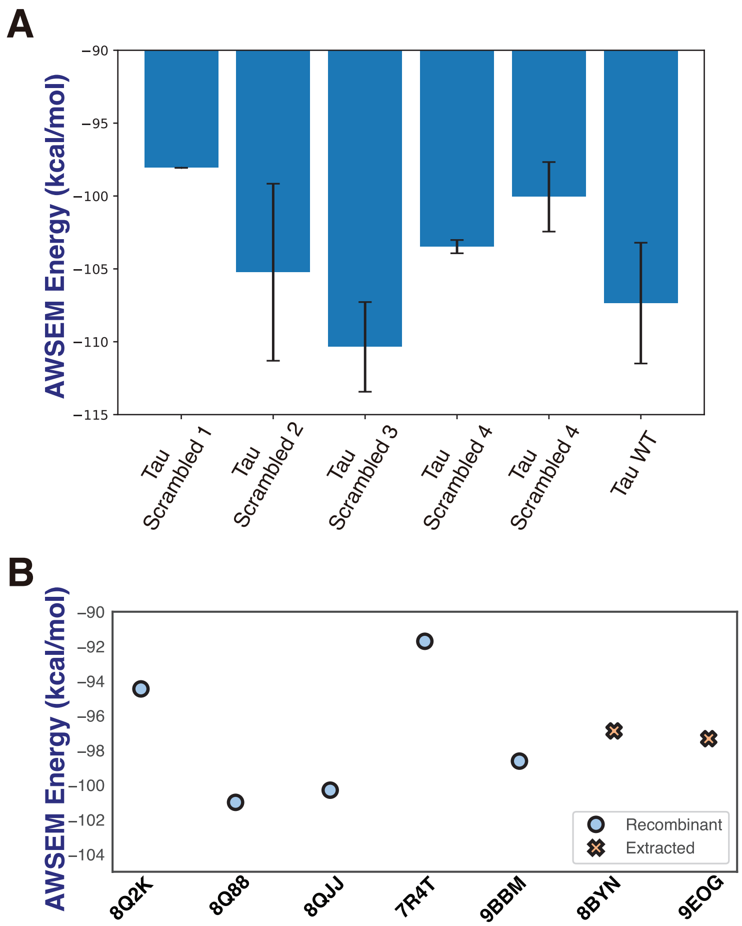

Figure S7: Comparing the elongation energetics of Tau from different sources and with scrambled sequences. A: In total, 20 predicted ribbons for each of 5 scrambled sequences were generated, and the binding energy values of each predicted ribbon was computed from AWSEM. The energetic distribution is illustrated as a barplot for each ensemble, and the error bars illustrate the standard deviations. B: In comparison, experimentally observed ribbons of Tau from different sources are also evaluated for their AWSEM energies. The colors indicate their corresponding experimental sources.

S3.4 Fibril polymorphism of scrambled α-Synuclein Polypeptides.

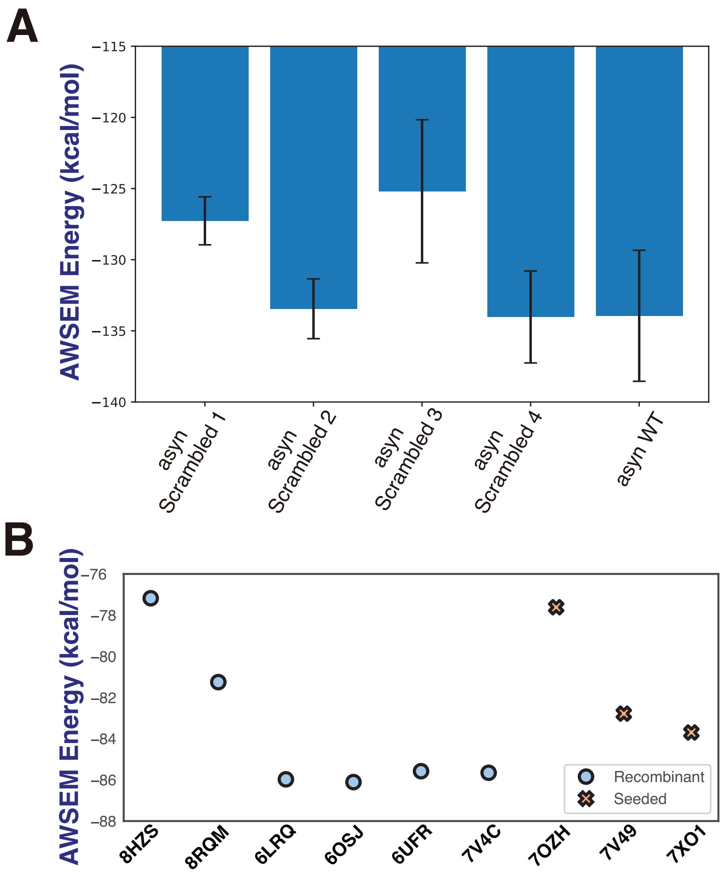

Figure S8: Comparing the elongation energetics of α-Synuclein from different sources and with scrambled sequences. A: In total, 20 predicted ribbons for each of 5 scrambled sequences were generated, and the binding energy values of each predicted ribbon was computed from AWSEM. The energetic distribution is illustrated as a barplot for each ensemble, and the error bars illustrate the standard deviations. B: In comparison, experimentally observed ribbons of α-Synuclein from different sources are also evaluated for their AWSEM energies. The colors indicate their corresponding experimental sources.

S3.5 Fibril polymorphism of TDP-43 Polypeptides.

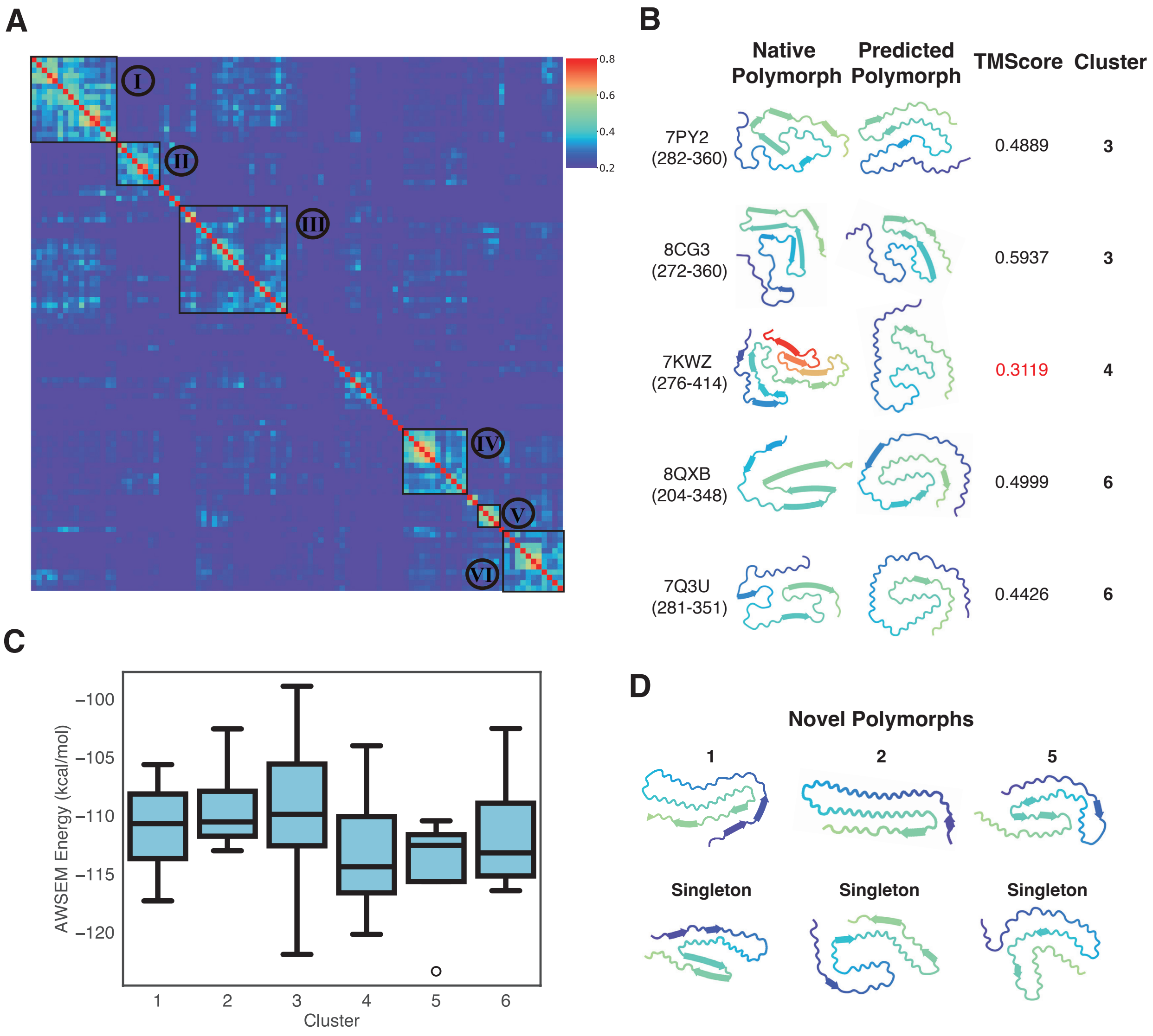

Figure S9: Polymorph landscape of the monomeric TDP-43 ribbon predicted by RibbonFold. A: Clustered polymorphs for 100 predictions of five TDP-43 peptides in a monomer protofilament, using mutual-Q as the metric for measuring structural similarity. One hundred predicted ribbon structures were hierarchically clustered and are shown in a heatmap on the left. The identified clusters are enclosed in black squares on the heatmap, and the centroid structure from each cluster is shown and colored according to the sequence index from blue (N-terminal) to red (C-terminal) on the right. B: The representative polymorph from each predicted cluster is shown in a 2D ribbon form, and their corresponding native polymorph is also found and shown in accompany. Some predicted polymorphs don't have a well-defined experimental hit, and are shown as singleton polymorphs. C: Potential energy values of different clusters computed from AWSEM. D: Representative novel polymorphs identified from 100 predictions, where these polymorphs do not have corresponding experimental hits.

S3.6 Polymorph landscape of Orb2, apCPEB-Q, Sup35 and Ure2p computed by RibbonFold

Orb2A is a prion-like RNA-binding protein found in Drosophila melanogaster (fruit flies) and plays a crucial role in synaptic plasticity and long-term memory formation. Orb2A is a member of the Cytoplasmic Polyadenylation Element-Binding (CPEB) protein family and exists in two isoforms: Orb2A and Orb2B. Orb2A?s ability to form amyloid-like aggregates is essential for maintaining persistent changes in synaptic strength, a process linked to memory consolidation. Our polymorph analysis of the 100 structures predicted by RibbonFold revealed 6 major clusters, encompassing 94% of the predictions (Fig S7).

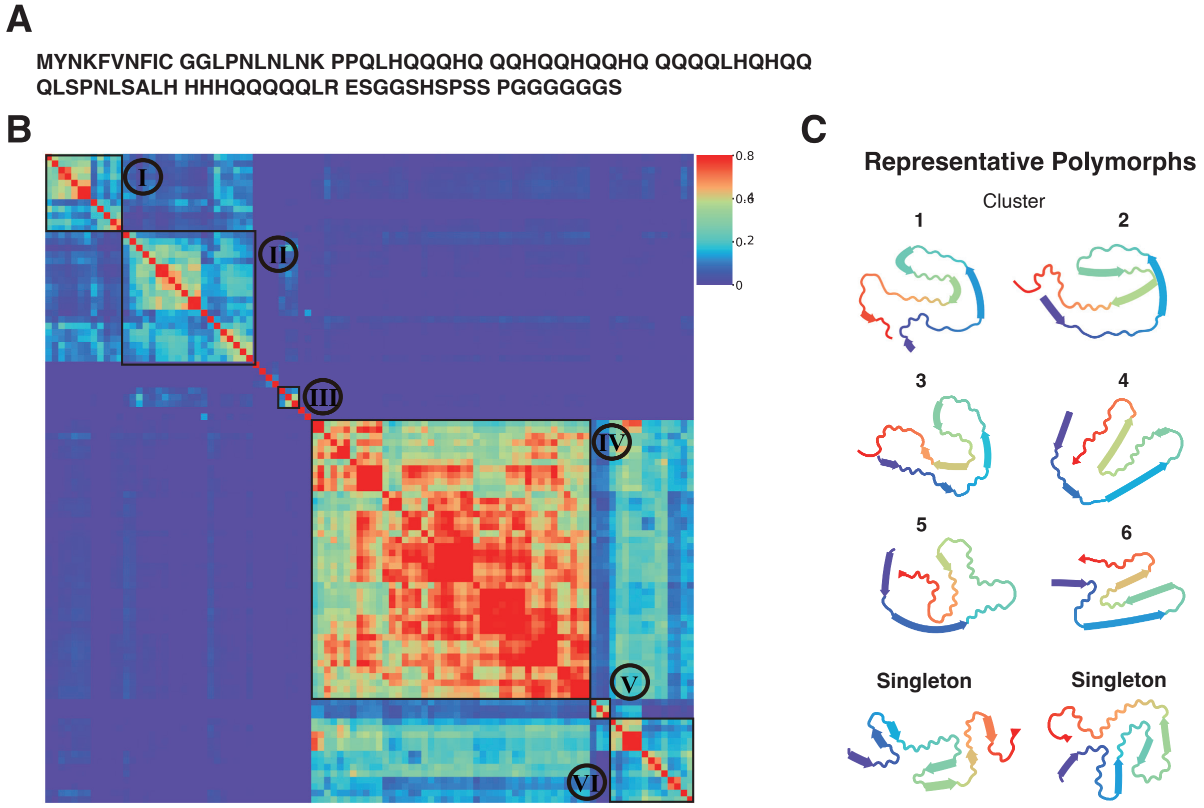

Figure S10: Polymorph landscape of the monomeric Orb2A$_{1-91}$ ribbon predicted by RibbonFold. A: The sequence of Orb2A's prion-like domain is illustrated. B: Clustered polymorphs for 100 predictions of five Orb2A peptides in a monomer protofilament, using mutual-Q as the metric for measuring structural similarity. One hundred predicted ribbon structures were hierarchically clustered and are shown in a heatmap on the left. The identified clusters are enclosed in black squares on the heatmap, and the centroid structure from each cluster is shown and colored according to the sequence index from blue (N-terminal) to red (C-terminal) on the right. C: The representative polymorph from each predicted cluster is shown in a 2D ribbon form.

ApCPEB (Aplysia CPEB) is a prion-like RNA-binding protein found in Aplysia californica (sea slug). It plays a pivotal role in synaptic plasticity and long-term memory storage by regulating local mRNA translation at synapses. ApCPEB aggregation is driven by prion-like domains (PLD). Our analysis of 100 predicted structures identified 6 main polymorphic clusters, covering 72% of all predictions.

A

**QSQSQQQQQQ TQQQQQQQQQ QQQQQHLQQV QQQQLLKQQQ QQAQQQQIQQ
QLLQQQQQKQ QLQQQQQQEQ LQQQQLQLQQ QLQQQLQH**

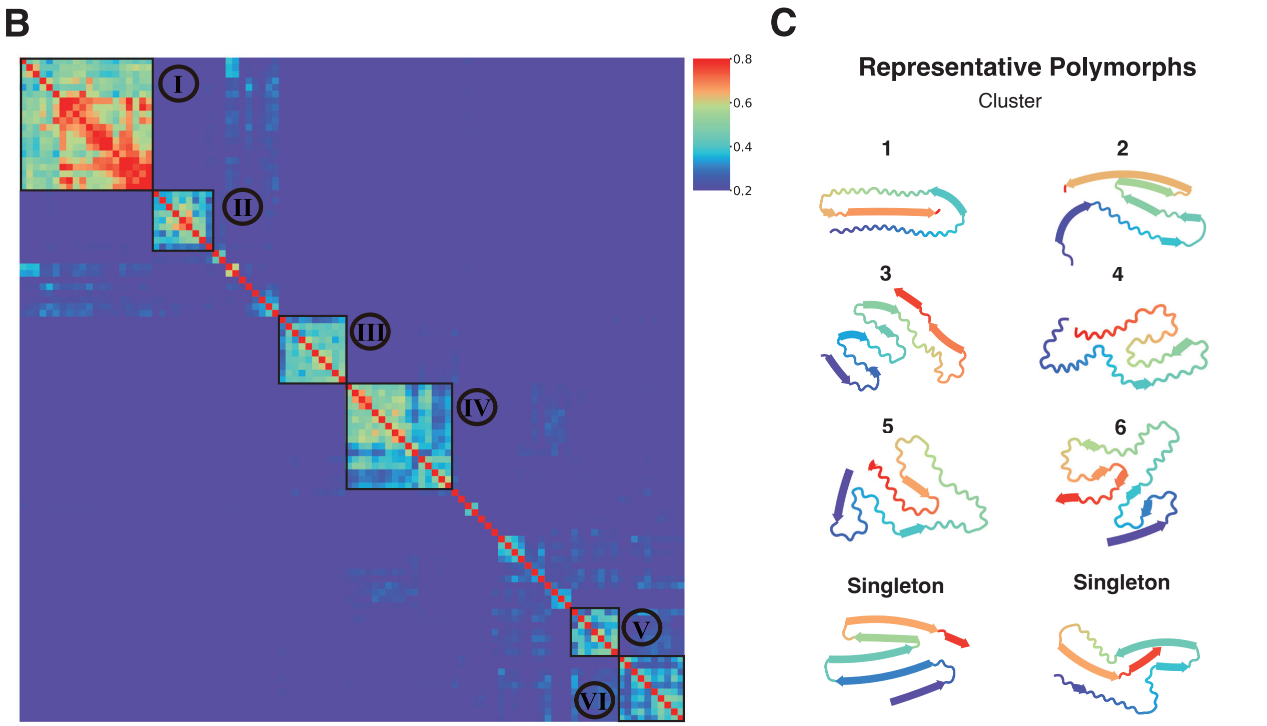

Figure S11: Polymorph landscape of the monomeric apCPEB ribbon predicted by RibbonFold. A: The sequence of apCPEB's prion-like domain is illustrated. B: Clustered polymorphs for 100 predictions of five apCPEB peptides in a monomer protofilament, using mutual-Q as the metric for measuring structural similarity. One hundred predicted ribbon structures were hierarchically clustered and are shown in a heatmap on the left. The identified clusters are enclosed in black squares on the heatmap, and the centroid structure from each cluster is shown and colored according to the sequence index from blue (N-terminal) to red (C-terminal) on the right. C: The representative polymorph from each predicted cluster is shown in a 2D ribbon form.

Sup35 is a yeast prion protein found in Saccharomyces cerevisiae. It is a translation termination factor (eRF3) that functions in ribosome-mediated release of polypeptides during protein synthesis. Sup35 can exist in two distinct states: a soluble, functional form and an aggregated, prion-like form called [PSI+]. The prion form [PSI+] affects translation termination fidelity, causing readthrough of stop codons and resulting in phenotypic diversity. Predictions for Sup35 yielded 2 clustered polymorphs, covering only 7% of all predictions. This diversity highlights the functional and prion-like versatility of Sup35, aligning with its known biological roles.

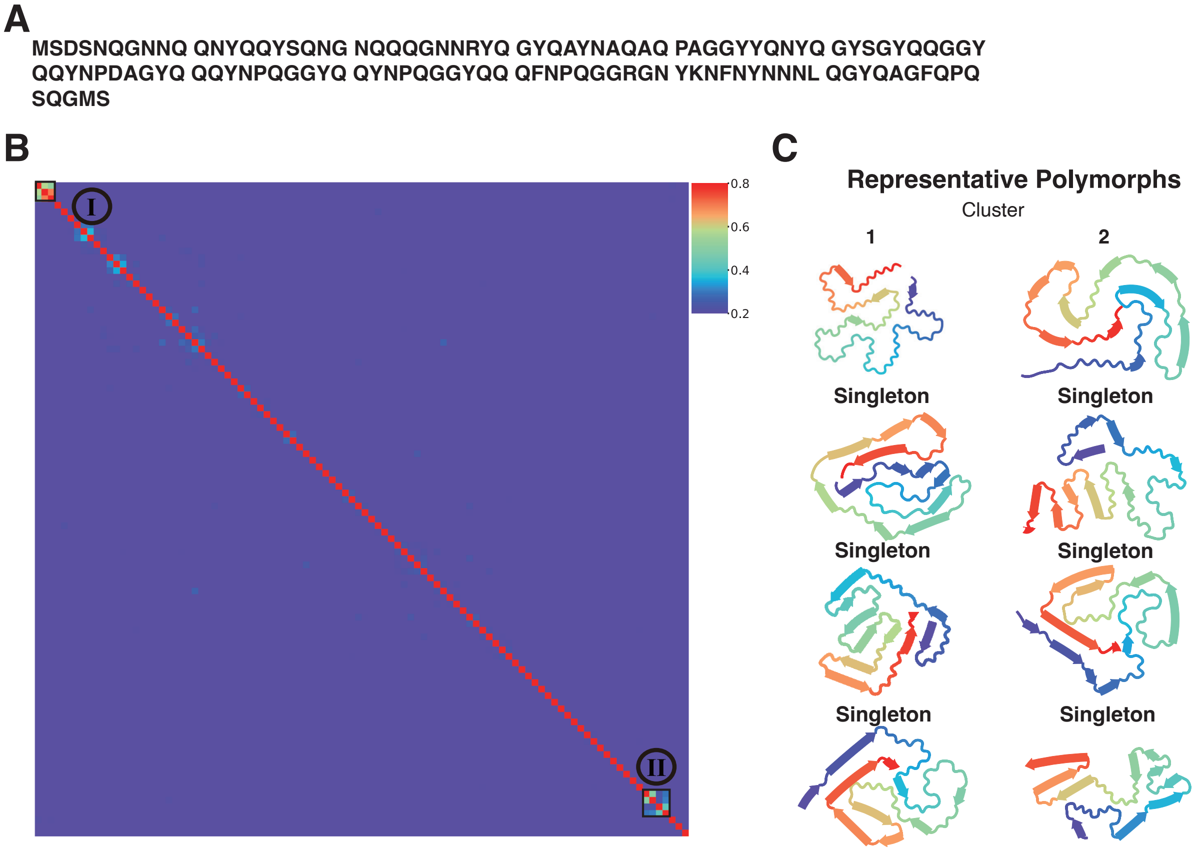

Figure S12: Polymorph landscape of the monomeric sup35$-NM$ ribbon predicted by RibbonFold. A: The sequence of Sup35's prion-like domain is illustrated. B: Clustered polymorphs for 100 predictions of five sup35$-NM$ peptides in a monomer protofilament, using mutual-Q as the metric for measuring structural similarity. One hundred predicted ribbon structures were hierarchically clustered and are shown in a heatmap on the left. The identified clusters are enclosed in black squares on the heatmap, and the centroid structure from each cluster is shown and colored according to the sequence index from blue (N-terminal) to red (C-terminal) on the right. C: The representative polymorph from each predicted cluster is shown in a 2D ribbon form.

Ure2p is another yeast prion protein found in Saccharomyces cerevisiae and is involved in nitrogen metabolism. In its prion form, called [URE3], Ure2p loses its regulatory function, allowing the expression of genes for alternative nitrogen source utilization. Analysis of Ure2p?s predicted structures revealed 6 major polymorphic clusters, covering 93% of 100 predictions.

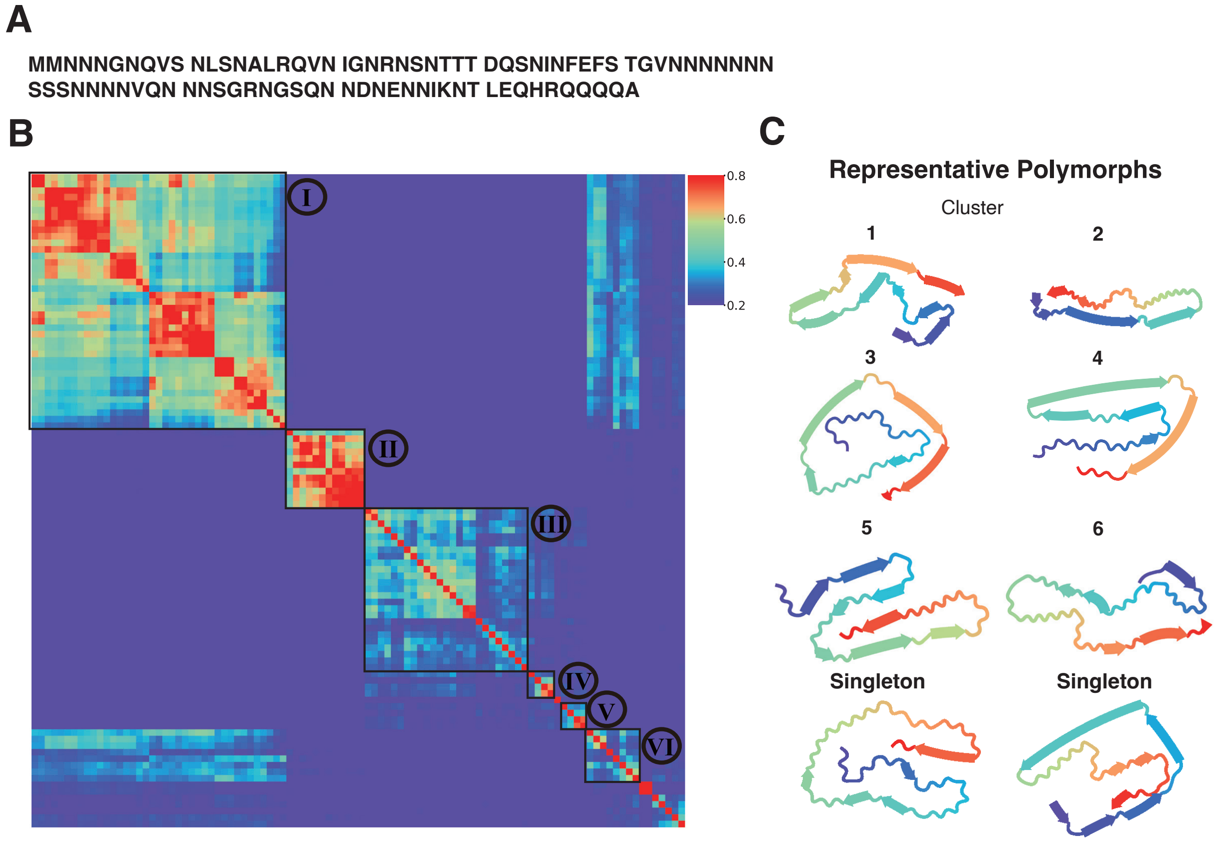

Figure S13: Polymorph landscape of the monomeric Ure2p$_{1-91}$ ribbon predicted by RibbonFold. A: The sequence of Ure2p's prion-like domain is illustrated. B: Clustered polymorphs for 100 predictions of five Ure2p peptides in a monomer protofilament, using mutual-Q as the metric for measuring structural similarity. One hundred predicted ribbon structures were hierarchically clustered and are shown in a heatmap on the left. The identified clusters are enclosed in black squares on the heatmap, and the centroid structure from each cluster is shown and colored according to the sequence index from blue (N-terminal) to red (C-terminal) on the right. C: The representative polymorph from each predicted cluster is shown in a 2D ribbon form.

S3.7 Experimental correspondence of predictions for sup35$-NM$

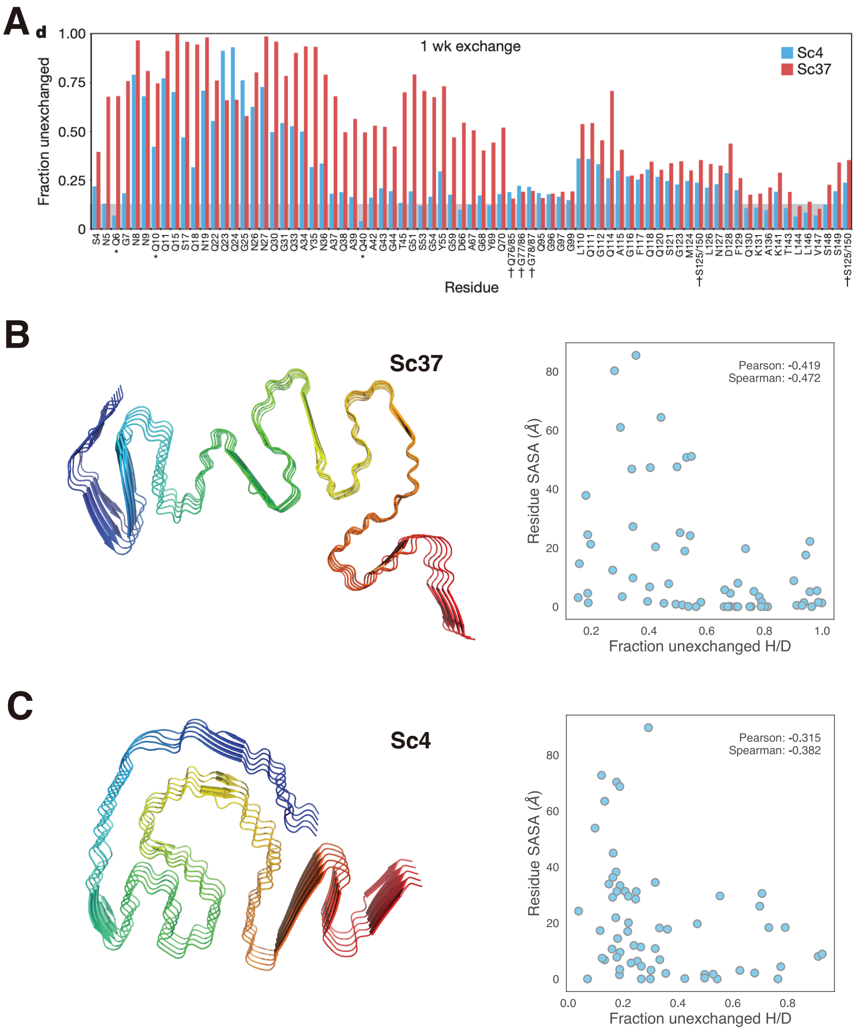

Figure S14: Comparing the predicted structures with Hydrogen/Deuterium exchange results. A: H/D exchange of Sc4 and Sc37 fibres obtained after incubation for 1 week[?]. B: The H/D exchange profile of Sc37 was compared with predictions made by RibbonFold, and the best matched prediction (solvent accessible surface area (SASA) of each residue v.s. unexchanged fraction) is shown on the left (each chain is colored from blue to red from N to C-termini). C: The H/D exchange profile of Sc4 was compared with predictions made by RibbonFold, and the best matched prediction is shown on the left (each chain is colored from blue to red from N to C-terminal).

S3.8 Representative structures from polyQ peptides of different lengths computed by RibbonFold

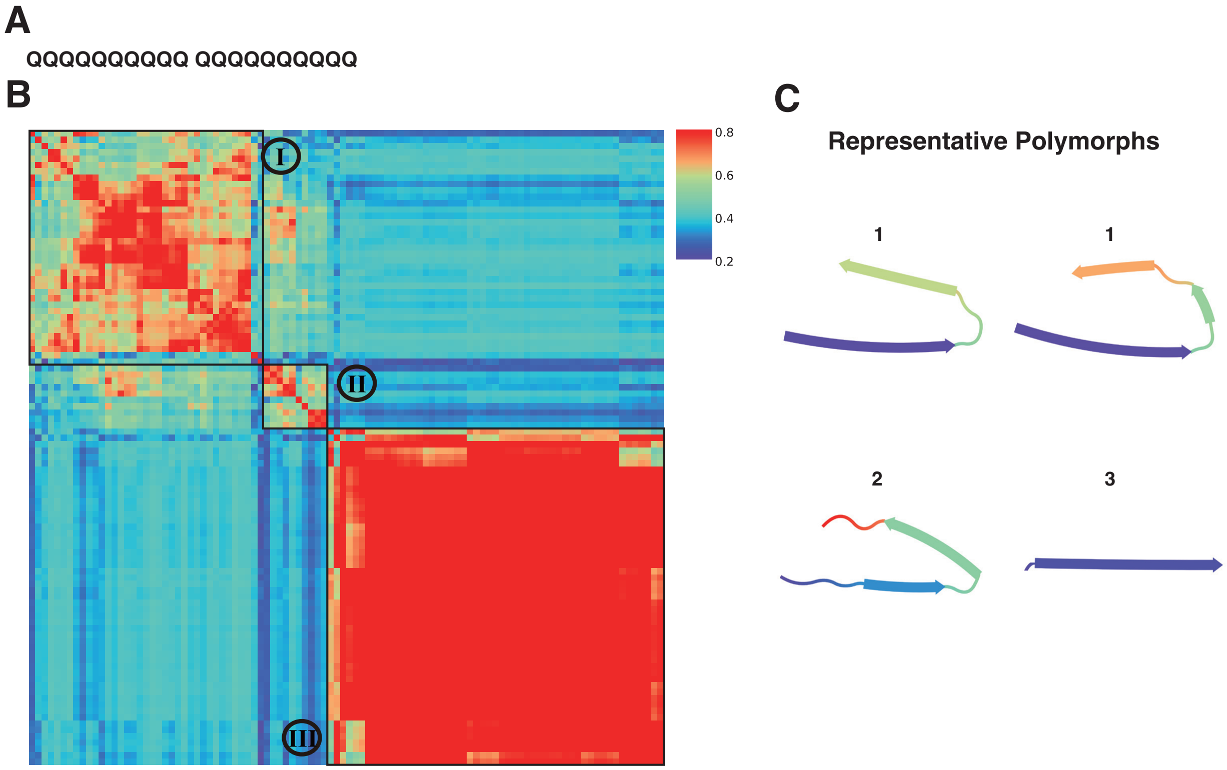

Figure S15: Polymorph landscape of the monomeric Q20 ribbon predicted by RibbonFold. A: The sequence of Q20 is illustrated. B: Clustered polymorphs for 100 predictions of five Q20 peptides in a monomer protofilament, using mutual-Q as the metric for measuring structural similarity. One hundred predicted ribbon structures were hierarchically clustered and are shown in a heatmap on the left. The identified clusters are enclosed in black squares on the heatmap, and the centroid structure from each cluster is shown and colored according to the sequence index from blue (N-terminal) to red (C-terminal) on the right. C: The representative polymorph from each predicted cluster is shown in a 2D ribbon form.

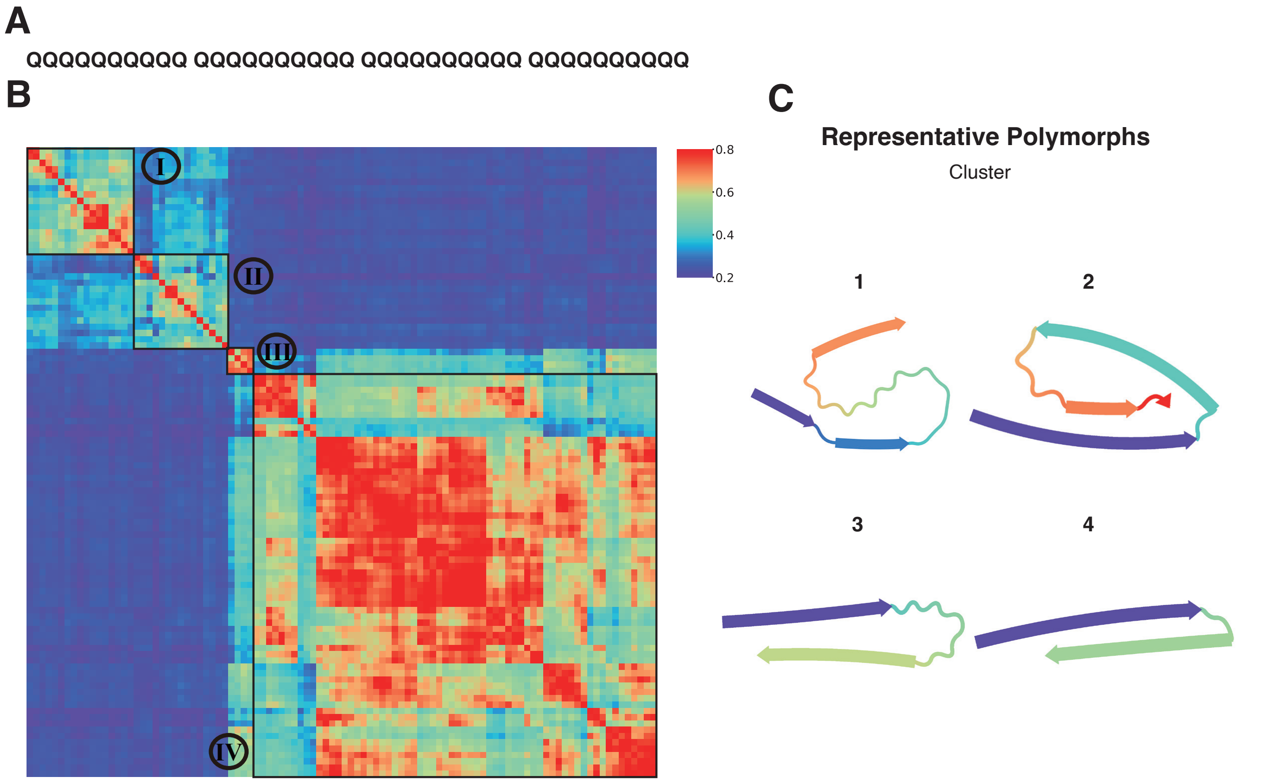

Figure S16: Polymorph landscape of the monomeric Q40 ribbon predicted by RibbonFold. A: The sequence of Q40 is illustrated. B: Clustered polymorphs for 100 predictions of five Q40 peptides in a monomer protofilament, using mutual-Q as the metric for measuring structural similarity. One hundred predicted ribbon structures were hierarchically clustered and are shown in a heatmap on the left. The identified clusters are enclosed in black squares on the heatmap, and the centroid structure from each cluster is shown and colored according to the sequence index from blue (N-terminal) to red (C-terminal) on the right. C: The representative polymorph from each predicted cluster is shown in a 2D ribbon form.

S4 Polymorph landscapes computed from various proteins by AlphaFold3-Server

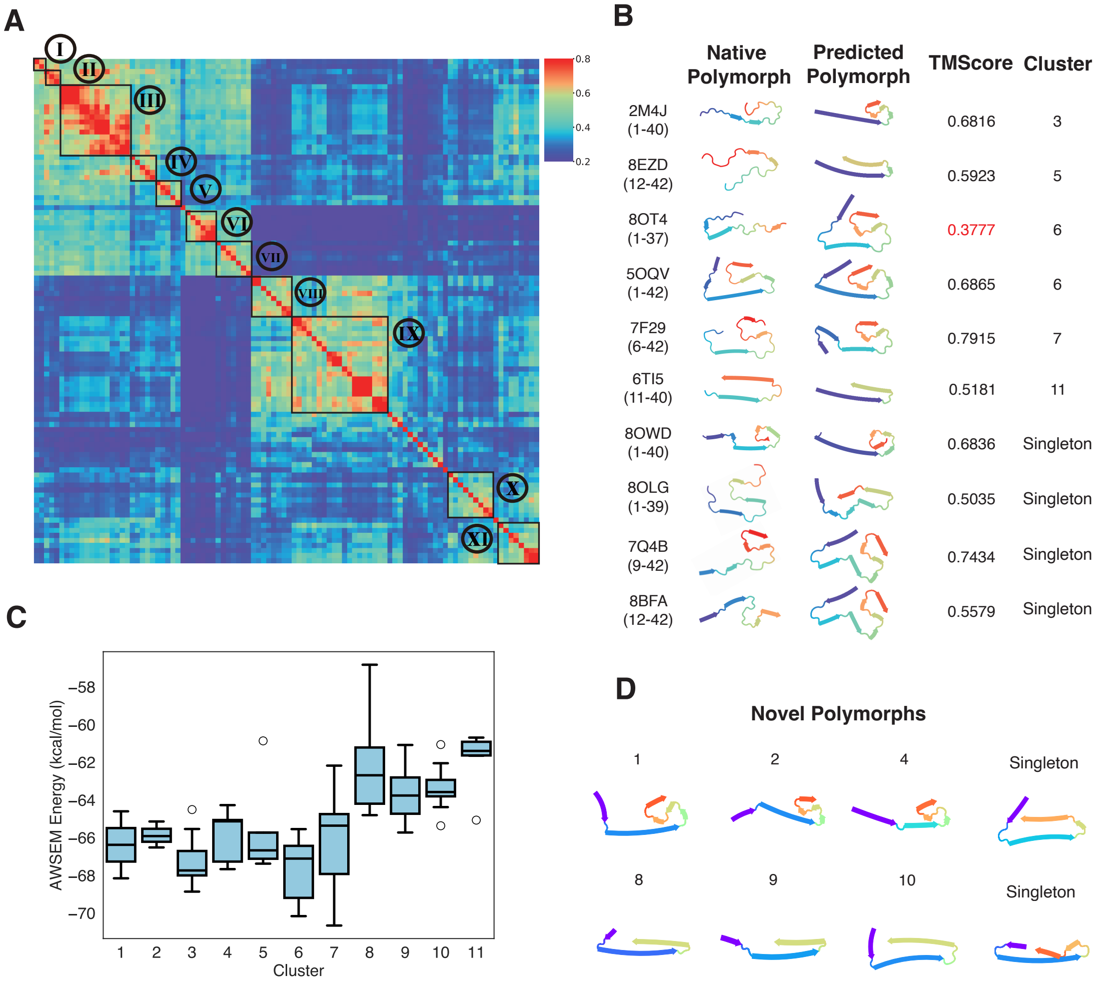

Figure S17: Polymorph landscape of the monomeric Aβ_{1-42} ribbon predicted by AlphaFold3-Server. A: Clustered polymorphs for 100 predictions of five Aβ_{1-42} peptides in a monomer protofilament, using mutual-Q as the metric for measuring structural similarity. One hundred predicted ribbon structures were hierarchically clustered and are shown in a heatmap on the left. The identified clusters are enclosed in black squares on the heatmap, and the centroid structure from each cluster is shown and colored according to the sequence index from blue (N-terminal) to red (C-terminal) on the right. B: The representative polymorph from each predicted cluster is shown in a 2D ribbon form, and their corresponding native polymorph is also found and shown in accompany. Some predicted polymorphs don't have a well-defined experimental hit, and are shown as singleton polymorphs. C: Potential energy values of different clusters computed from both Rosetta and AWSEM. D: Representative novel polymorphs identified from 100 predictions, where these polymorphs do not have corresponding experimental hits.

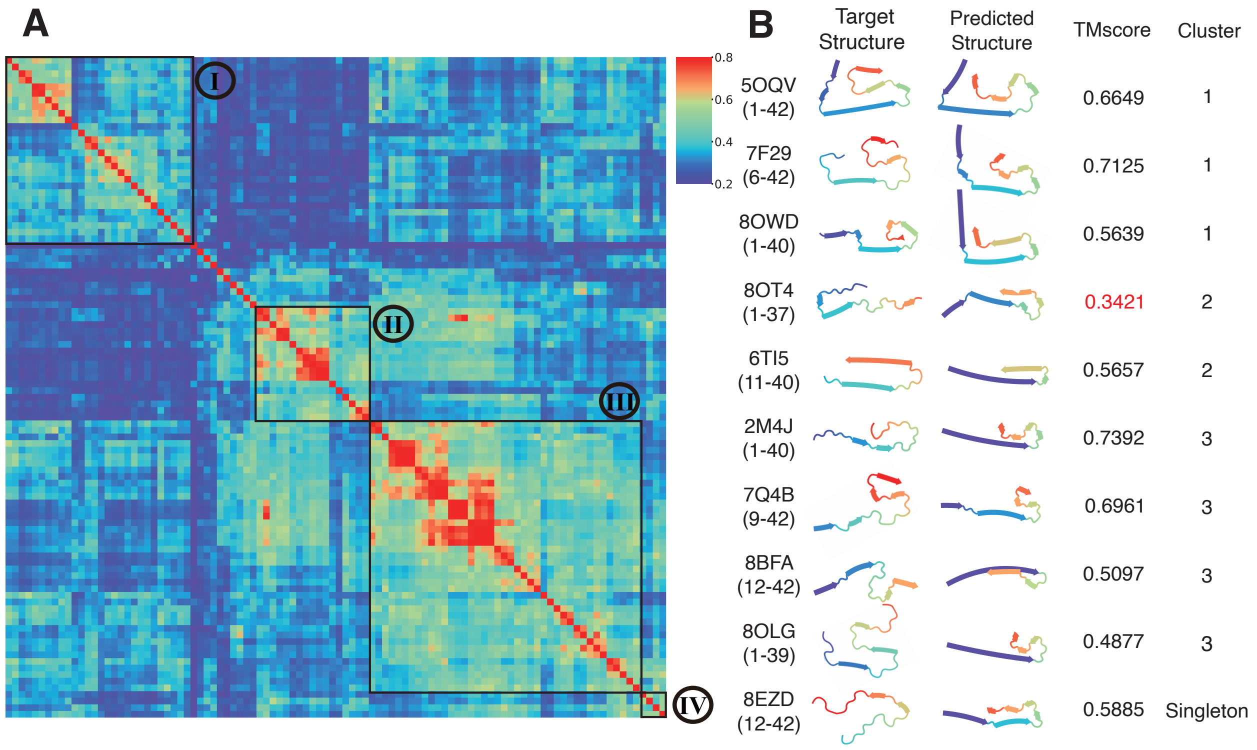

Figure S18: Polymorph landscape of the monomeric Aβ_{1-40} ribbon predicted by AlphaFold3-Server. A: Clustered polymorphs for 100 predictions of five Aβ_{1-40} peptides in a monomer protofilament, using mutual-Q as the metric for measuring structural similarity. One hundred predicted ribbon structures were hierarchically clustered and are shown in a heatmap on the left. The identified clusters are enclosed in black squares on the heatmap, and the centroid structure from each cluster is shown and colored according to the sequence index from blue (N-terminal) to red (C-terminal) on the right. B: The representative polymorph from each predicted cluster is shown in a 2D ribbon form, and their corresponding native polymorph is also found and shown in accompany. Some predicted polymorphs don't have a well-defined experimental hit, and are shown as singleton polymorphs.

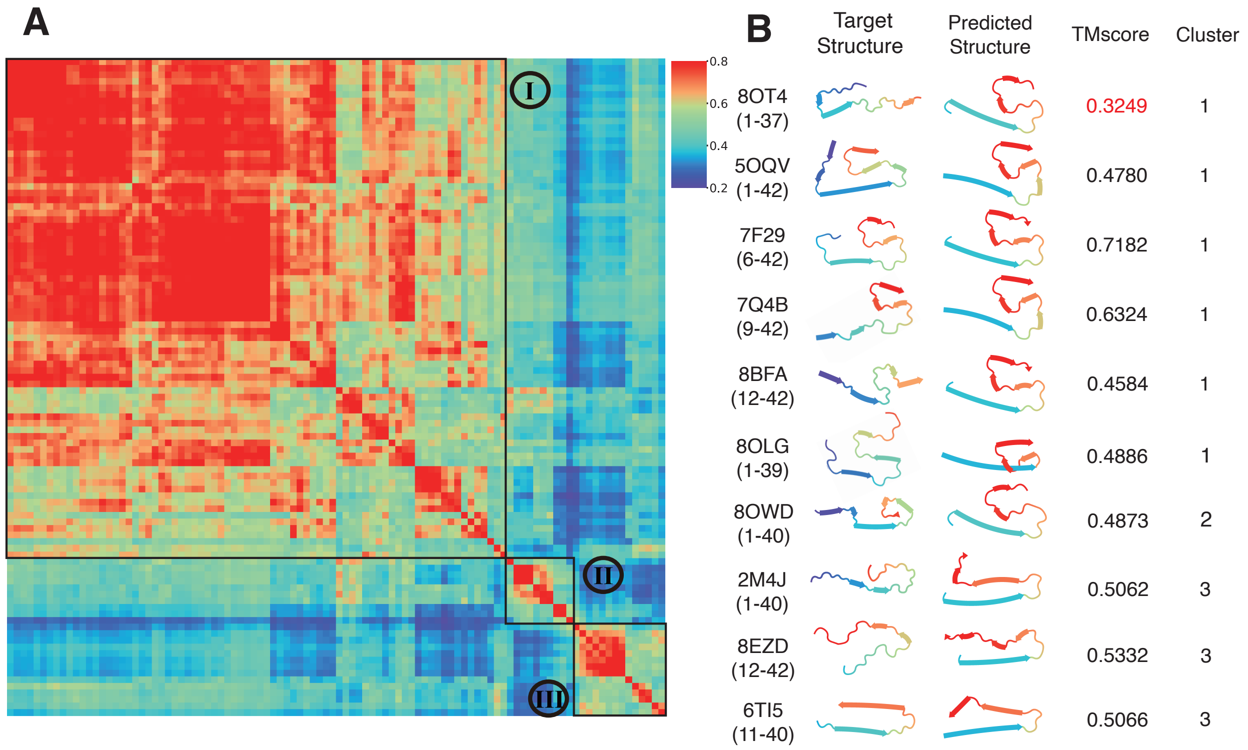

Figure S19: Polymorph landscape of the monomeric Aβ_{12-42} ribbon predicted by AlphaFold3-Server. A: Clustered polymorphs for 100 predictions of five Aβ_{12-42} peptides in a monomer protofilament, using mutual-Q as the metric for measuring structural similarity. One hundred predicted ribbon structures were hierarchically clustered and are shown in a heatmap on the left. The identified clusters are enclosed in black squares on the heatmap, and the centroid structure from each cluster is shown and colored according to the sequence index from blue (N-terminal) to red (C-terminal) on the right. B: The representative polymorph from each predicted cluster is shown in a 2D ribbon form, and their corresponding native polymorph is also found and shown in accompany. Some predicted polymorphs don't have a well-defined experimental hit, and are shown as singleton polymorphs.

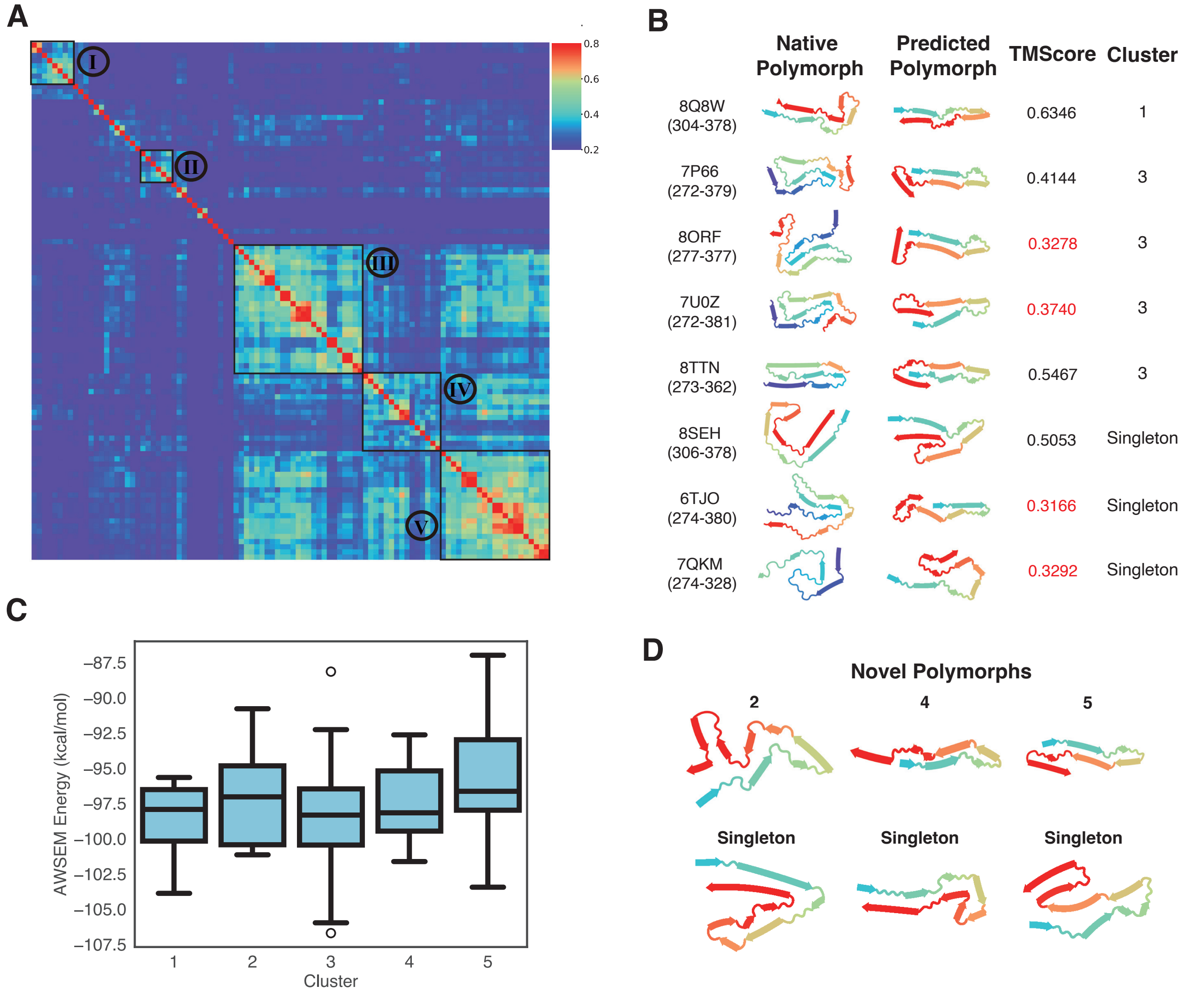

Figure S20: Polymorph landscape of the monomeric Tau ribbon predicted by AlphaFold3-Server. A: Clustered polymorphs for 100 predictions of five Tau peptides in a monomer protofilament, using mutual-Q as the metric for measuring structural similarity. One hundred predicted ribbon structures were hierarchically clustered and are shown in a heatmap on the left. The identified clusters are enclosed in black squares on the heatmap, and the centroid structure from each cluster is shown and colored according to the sequence index from blue (N-terminal) to red (C-terminal) on the right. B: The representative polymorph from each predicted cluster is shown in a 2D ribbon form, and their corresponding native polymorph is also found and shown in accompany. Some predicted polymorphs don't have a well-defined experimental hit, and are shown as singleton polymorphs. C: Potential energy values of different clusters computed from AWSEM. D: Representative novel polymorphs identified from 100 predictions, where these polymorphs do not have corresponding experimental hits.

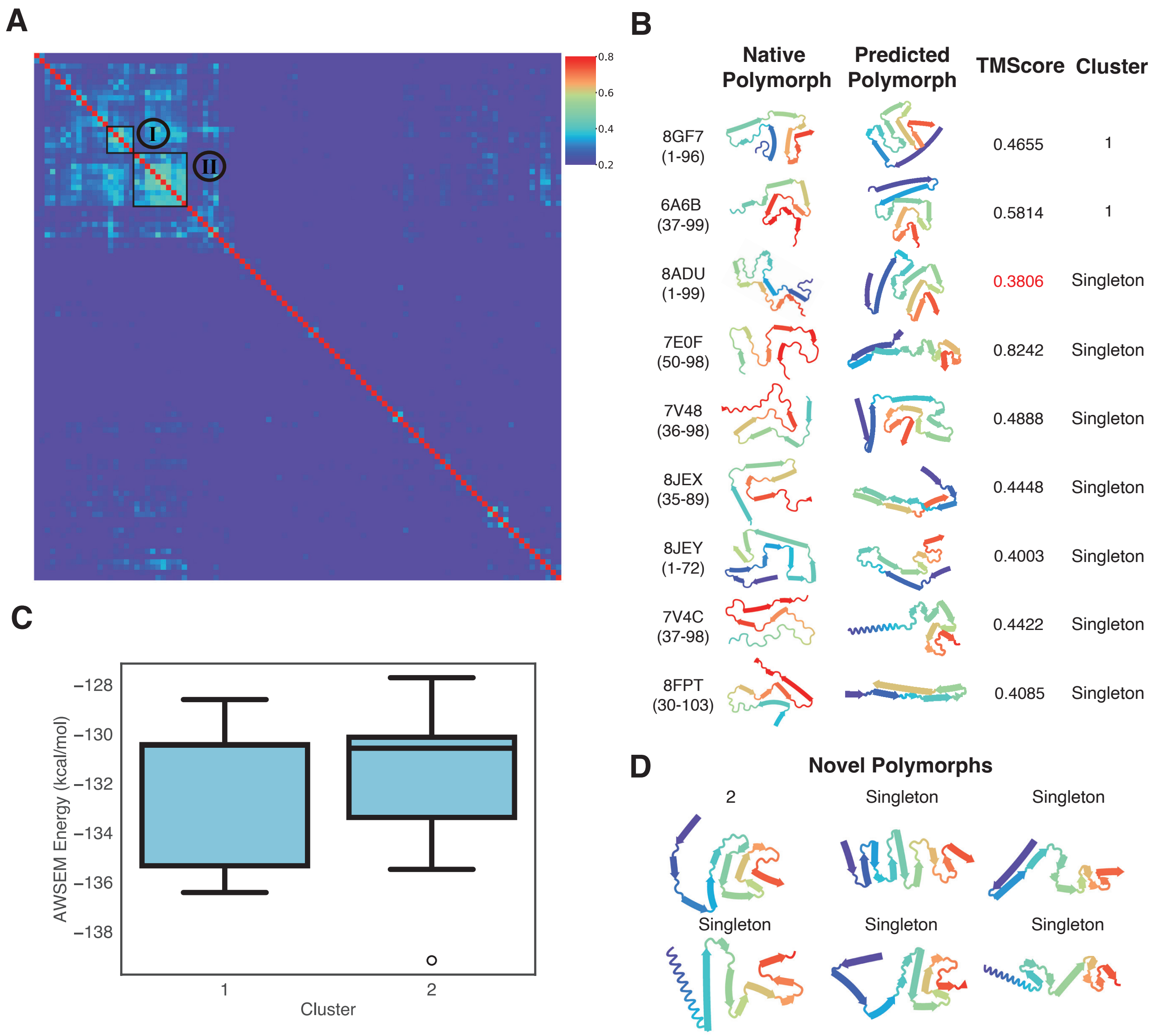

Figure S21: Polymorph landscape of the monomeric α-synuclein ribbon predicted by AlphaFold3-Server. A: Clustered polymorphs for 100 predictions of five α-synuclein peptides in a monomer protofilament, using mutual-Q as the metric for measuring structural similarity. One hundred predicted ribbon structures were hierarchically clustered and are shown in a heatmap on the left. The identified clusters are enclosed in black squares on the heatmap, and the centroid structure from each cluster is shown and colored according to the sequence index from blue (N-terminal) to red (C-terminal) on the right. B: The representative polymorph from each predicted cluster is shown in a 2D ribbon form, and their corresponding native polymorph is also found and shown in accompany. Some predicted polymorphs don't have a well-defined experimental hit, and are shown as singleton polymorphs. C: Potential energy values of different clusters computed from AWSEM. D: Representative novel polymorphs identified from 100 predictions, where these polymorphs do not have corresponding experimental hits.

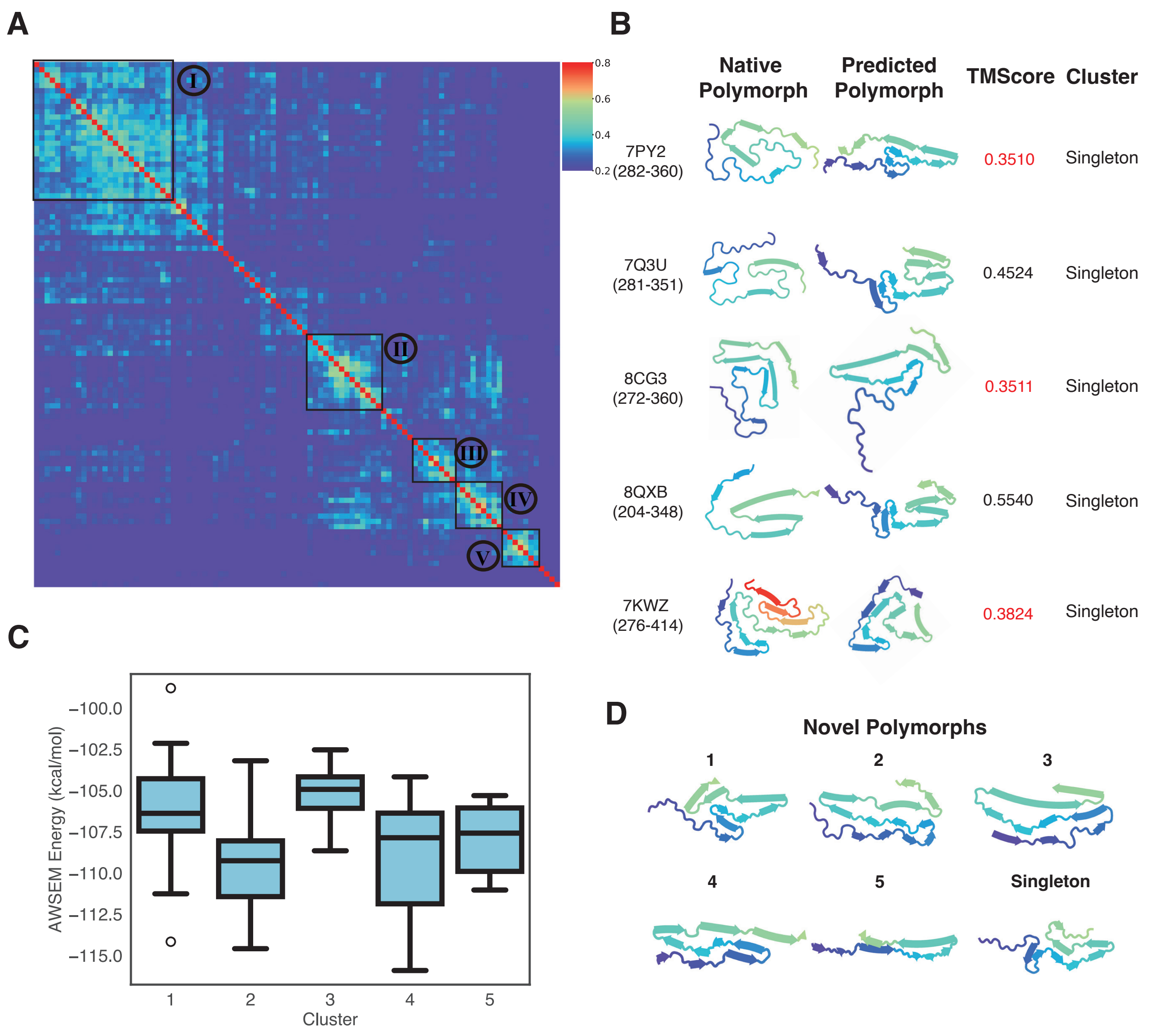

Figure S22: Polymorph landscape of the monomeric TDP-43 ribbon predicted by AlphaFold3-Server. A: Clustered polymorphs for 100 predictions of five TDP-43 peptides in a monomer protofilament, using mutual-Q as the metric for measuring structural similarity. One hundred predicted ribbon structures were hierarchically clustered and are shown in a heatmap on the left. The identified clusters are enclosed in black squares on the heatmap, and the centroid structure from each cluster is shown and colored according to the sequence index from blue (N-terminal) to red (C-terminal) on the right. B: The representative polymorph from each predicted cluster is shown in a 2D ribbon form, and their corresponding native polymorph is also found and shown in accompany. Some predicted polymorphs don't have a well-defined experimental hit, and are shown as singleton polymorphs. C: Potential energy values of different clusters computed from AWSEM. D: Representative novel polymorphs identified from 100 predictions, where these polymorphs do not have corresponding experimental hits.
